# TaPPR13, a Pentatricopeptide Repeat Protein Gene Activated by TaBZR2, Confers Drought Stress Tolerance by Enhancing the Antioxidant Defense System and Promoting Retrograde Signaling in Wheat (*Triticum aestivum*)

**DOI:** 10.1002/advs.202502984

**Published:** 2025-06-29

**Authors:** Ze‐Hao Hou, Wei‐Jun Zheng, Lei Zheng, Jing‐Yue Wang, Shuang‐Xi Zhang, Ji‐Tong Wei, Shu‐Hui Yang, Yuan‐Chen Jiao, Wen‐Jing Cheng, Tai‐Fei Yu, Xiao‐Fei Ma, Jing‐Na Ru, Yong‐Wei Liu, Xin‐You Cao, Jun Chen, Yong‐Bin Zhou, Ming Chen, Li‐Hui Li, You‐Zhi Ma, Xiao‐Jun Nie, Zhao‐Shi Xu

**Affiliations:** ^1^ State Key Laboratory of Crop Gene Resources and Breeding Institute of Crop Sciences Chinese Academy of Agricultural Sciences (CAAS) Beijing 100081 China; ^2^ State Key Laboratory for Crop Stress Resistance and High‐Efficiency Production College of Agronomy Northwest Agricultural and Forestry University Yangling 712100 China; ^3^ Institute of Crop Science Ningxia Academy of Agriculture and Forestry Sciences Yongning 750105 China; ^4^ Institute of Wheat Research The Industrial Crop Institute Shanxi Agricultural University Taiyuan 030000 China; ^5^ Institute of Biotechnology and Food Science Hebei Academy of Agriculture and Forestry Sciences/Hebei Key Laboratory of Drought‐Alkali Tolerance in Wheat Cangzhou 050051 China; ^6^ National Engineering Laboratory for Wheat and Maize/Key Laboratory of Wheat Biology and Genetic Improvement Crop Research Institute Shandong Academy of Agricultural Sciences Jinan 250100 China; ^7^ National Nanfan Research Institute (Sanya) Chinese Academy of Agricultural Sciences/Seed Industry Laboratory Sanya 572024 China

**Keywords:** chloroplast, drought tolerance, GWAS, ROS, retrograde signaling

## Abstract

The wheat (*Triticum aestivum*) brassinazole‐resistant 2 (*TaBZR2*) gene is identified as significantly associated with drought tolerance by genome‐wide association study (GWAS), and a chloroplast pentatricopeptide repeat (PPR) protein gene *TaPPR13* functioned as a positive drought stress regulator downstream of TaBZR2. Overexpression of *TaPPR13* enhanced the antioxidative defense system, whereas knockdown of *TaPPR13* led to the accumulation of reactive oxygen species (ROS) and caused abnormalities in chloroplast thylakoids under drought stress conditions. RNA‐seq analysis showed that overexpression of *TaPPR13* significantly upregulated the expression of nuclear‐encoded genes involved in ROS scavenging and the abscisic acid (ABA) signaling pathway. Furthermore, TaPPR13 interacted with TaAOR1 and TaSIG5 to facilitate detoxification and regulate chloroplast gene expression, thereby enhancing drought tolerance. Overexpression of *TaPPR13* and *TaAOR1* mediated stomatal closure to reduce water loss, improving photosynthetic capacity and conferring a yield advantage under drought stress. These findings show that TaPPR13 promotes retrograde signaling to alter nuclear gene expression, with the TaBZR2‐TaPPR13‐TaAOR1/TaSIG5 module representing a novel signaling pathway that likely plays a pivotal role in drought stress response.

## Graphical abstract

*TaPPR13*, a PPR protein family gene in wheat is activated by TaBZR2 to enhance drought stress tolerance and photosynthetic capacity. It achieves this by interacting with TaAOR1 and TaSIG5 to improve antioxidant defense and regulate chloroplast gene expression.

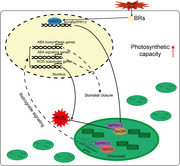

## 1 Introduction

Wheat is cultivated globally, particularly in arid and semiarid regions.^[^ ^1^, ^2^ ^]^ Consequently, drought stress is the most common environmental factor limiting wheat cultivation and productivity.^[^ ^3^, ^4^ ^]^ Plants have evolved various mechanisms to detect stress signals and adjust to unfavorable circumstances,^[^ ^5^, ^6^ ^]^ with increasing evidence that chloroplasts play a critical role in stress response and adaptation.^[^ ^7^, ^8^, ^9^ ^]^ Under adverse conditions, reactive oxygen species (ROS) are produced in the chloroplasts,^[^ ^9^, ^10^ ^]^ where they function as a retrograde signal to alter the transcriptome network, enabling adaptation to extreme stress.^[^ ^5^, ^9^ ^]^ ROS signaling boosts antioxidative defense by inducing expression of genes that encode antioxidant enzymes and abiotic stress response proteins.^[^ ^9^, ^10^, ^11^, ^12^, ^13^ ^]^ ROS also serves as a secondary messenger to participate in abscisic acid (ABA) signaling.^[^ ^12^, ^14^, ^15^ ^]^ AtGPX3, a glutathione peroxidase 3 protein in Arabidopsis functions as a general ROS scavenger and redox transducer in ABA and drought stress signaling.^[^ ^15^, ^16^ ^]^ AtCPK8, a calcium‐dependent protein kinase 8, is induced by drought stress and functions in ABA‐mediated stomatal regulation through phosphorylating AtCAT3 and modulating its activity.^[^ ^17^, ^18^ ^]^

Pentatricopeptide repeat (PPR) proteins, which constitute one of the largest protein families in plants,^[^ ^19^, ^20^, ^21^ ^]^ participate in a number of biological processes,^[^ ^22^, ^23^, ^24^ ^]^ such as posttranscriptional gene regulation,^[^ ^24^, ^25^ ^]^ chloroplast development,^[^ ^23^, ^26^ ^]^ male fertility,^[^ ^27^, ^28^ ^]^ and retrograde signaling.^[^ ^29^, ^30^ ^]^ An increasing number of studies has highlighted the role of PPR proteins in response to abiotic stress.^[^ ^31^, ^32^, ^33^ ^]^ The chloroplast‐localized PPR protein GUN1 regulates gene expression and function as an integrator of retrograde signals.^[^ ^29^, ^30^ ^]^ Loss of function in the mitochondrially localized PPR protein PGN caused increased ROS production and hypersensitivity to salt stress.^[^ ^31^ ^]^ Overexpression of *GmPPR4* conferred drought tolerance by enhancing ROS scavenging in soybean,^[^ ^34^ ^]^ whereas knockdown of PPR protein gene *PPS1* increased ROS production and showed hypersensitivity to abiotic stress.^[^ ^21^ ^]^

Although previous analyses indicated the importance of PPR proteins in response to abiotic stress, little is known about their functions in wheat under water deficit conditions. In this study, TaBZR2, a positive drought stress tolerance transcription factor (TF), was identified by genome‐wide association study (GWAS). *TaPPR13* was activated by TaBZR2 and enhanced drought tolerance by facilitating ABA‐mediated stomatal movement. Additionally, we demonstrate that TaAOR1 and TaSIG5, interact with TaPPR13 to enhance ROS scavenging by altering the expression of chloroplast genes. These findings provide new insights for modulating wheat drought tolerance through the TaBZR2‐TaPPR13‐TaAOR1/TaSIG5 regulatory module.

## 2 Results

### 2.1 TaBZR2, a Positive Drought Tolerance TF, Promotes Expression of TaPPR13

**Figure 1:**
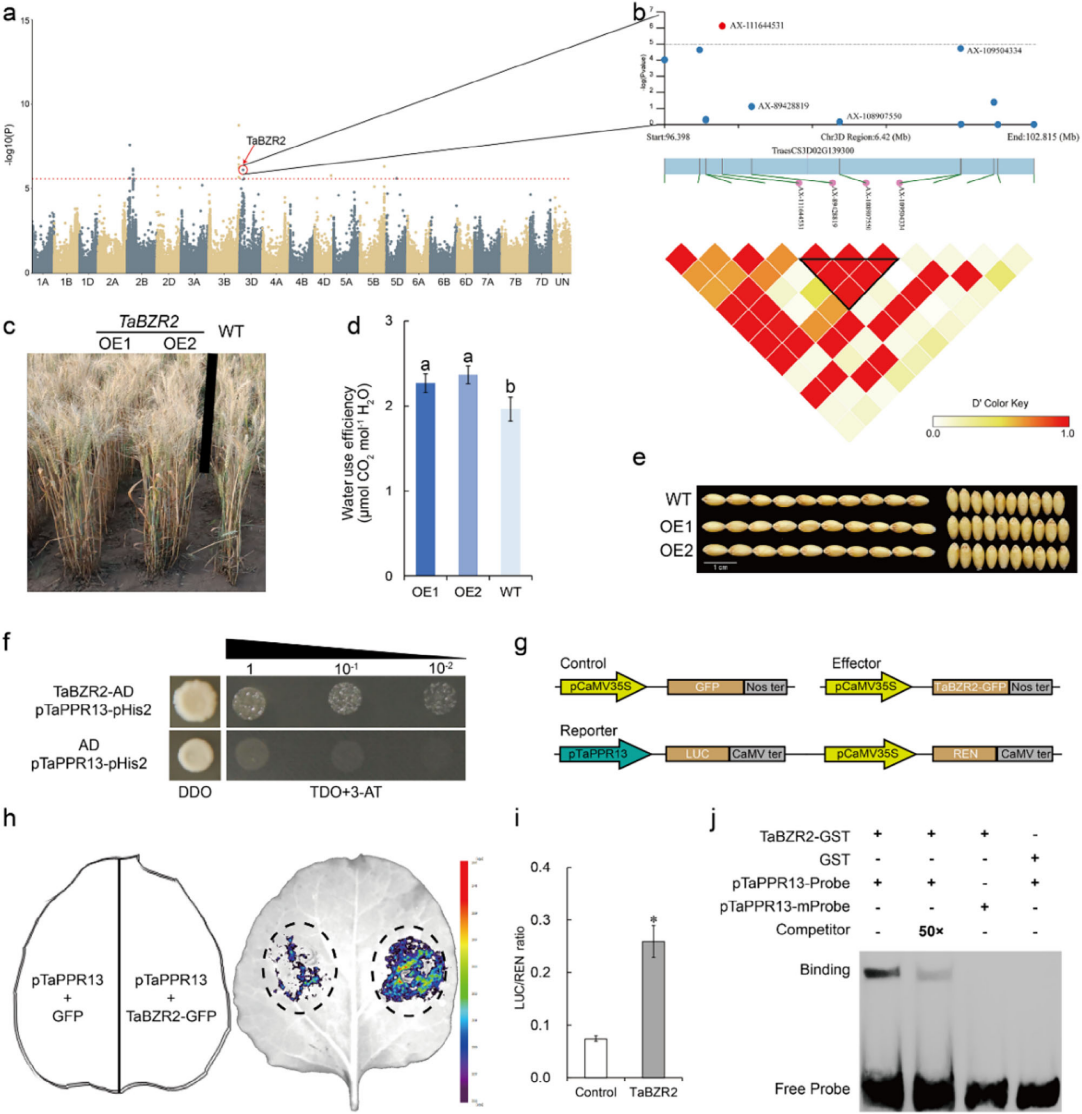
TaBZR2 was significantly associated with drought tolerance. (a) GWAS for drought tolerance in wheat seedlings. (b) Association mapping and pairwise LD analysis of *TaBZR2*. (c) Phenotypes of WT and *TaBZR2* transgenic wheat lines under drought stress in the field. (d) Water use efficiency of *TaBZR2*‐OE and WT plants under drought stress conditions. Values are means ± SD (*p* < 0.05, *n* = 12, one‐way ANOVA, Tukey's HSD test). (e) Grain length and width phenotypes of transgenic lines and WT plants. (f) Yeast one‐hybrid assay verifying interaction between TaBZR2 protein and *TaPPR13* promoter. (g–i) Transient luciferase (LUC) reporter assay showing that LUC activity driven by the *TaPPR13* promoter was markedly increased by the presence of TaBZR2 protein in tobacco leaves. Values are means ± SD (*p* < 0.05, *n* = 4, Student's *t*‐test). (j) EMSA assay showing that TaBZR2 can specifically bind to the *TaPPR13* promoter in vitro.

We determined the drought tolerance of 282 lines comprising a panel of 198 accessions introduced from the International Center for Research in Dryland Agriculture (ICARDA) and 84 genotypes from the Chinese Wheat Mini‐core Collection (Table S1, Supporting Information). The wheat 660K SNP array was used to identify genetic loci underlying tolerance drought stress. We noticed that *TaBZR2* (*TraesCS3D02G139300*), a positive drought tolerance TF gene identified in our previous study,^[^ ^35^ ^]^ was significantly associated with drought tolerance (**Figure** **1**a,b). We analyzed natural variation in the genomic region of *TaBZR2* to further explore its functional significance. We found an InDel (insertion‐deletion) in the 3′ UTR of the gene, and identified six haplotypes: Hap I and Hap II alleles were the most frequent haplotypes, whereas Hap III, Hap IV, Hap V, and Hap VI occurred at relatively lower frequencies (Figure S1a, Supporting Information). Further examination of the association between the major haplotypes (Hap I and Hap II) showed that total root length, aboveground fresh weight, root fresh weight, and total fresh weight of accessions carrying Hap II were significantly higher than those of accessions carrying Hap I (Figure S1b–e, Supporting Information). Furthermore, RT‐qPCR analysis showed that genotypes with *TaBZR2*‐Hap II had relatively higher expression levels than those with *TaBZR2*‐Hap I (Figure S1f, Supporting Information), suggesting that higher expression levels of *TaBZR2* contributed to increased wheat drought stress tolerance. *TaBZR2*‐OE plants, which were generated in our previous study,^[^ ^35^ ^]^ were subjected to standard field conditions to investigate the genetic effects of *TaBZR2*. Under water‐limited conditions, the water use efficiency (WUE) and plant agronomic traits in *TaBZR2*‐OE plants were greater than those of wild‐type (WT) plants (Figure 1c–e; Figure S2, Supporting Information). These data suggested that TaBZR2 play an important role in tolerance to drought stress, and indicated that the molecular mechanism by which TaBZR2 controls drought tolerance should be further investigated. Transcriptome analysis showed that expression of *TaPPR13* was up‐regulated in *TaBZR2*‐OE plants under drought stress,^[^ ^35^ ^]^ and comparative transcriptome analysis^[^ ^36^ ^]^ indicated that the *TaPPR13* gene was induced by drought stress, and ABA and BR treatments (Figure S3a,b, Supporting Information). We hypothesized that the expression of *TaPPR13* gene might be regulated by TaBZR2. Transcript factor binding site analysis revealed eight E‐box *cis*‐elements on the promoter of *TaPPR13* (Figure S4, Supporting Information). Though yeast one‐hybrid (Y1H) assay, we confirmed that TaBZR2 binds to the promoter of *TaPPR13* (Figure 1f). Regulation of TaBZR2 on the *TaPPR13* gene was further validated through transient luciferase (LUC) reporter assays in tobacco leaves. LUC activity driven by the *TaPPR13* promoter was markedly enhanced in the presence of the TaBZR2 protein (Figure 1g–i). Moreover, EMSA assays showed that TaBZR2 directly and specifically bound to biotin‐labeled probes generated with *TaPPR13* promoter fragments containing E‐box *cis*‐elements (Figure 1j). These results indicated that TaBZR2 positively activates expression of *TaPPR13* by binding to the E‐box *cis*‐elements.

*TaPPR13*, consists of a single 1833 bp exon encoding a putative 68.3 kDa protein with 13 PPR motifs (Figure S3c,d, Supporting Information). Phylogenetic analysis revealed that the TaPPR13 protein is closely related to OsWSL5 (Figure S3e, Supporting Information), a chloroplast‐targeted PPR protein that is crucial for chloroplast development.^[^ ^37^ ^]^ Tissue‐specific expression analysis showed that the *TaPPR13* gene is highly expressed in leaves (Figure S5a, Supporting Information), and RT‐qPCR and GUS staining showed that the transcript level of *TaPPR13* was induced by PEG6000, BR and ABA treatments (Figure S5b–e, Supporting Information). Subcellular localization assays showed that the TaPPR13‐GFP fusion protein was localized in the chloroplast (Figure S5f, Supporting Information).

### 2.2 TaPPR13 Positively Regulates Drought Stress Tolerance in Wheat

To verify the function of TaPPR13 on drought stress tolerance, we generated *TaPPR13*‐overexpression (OE) and *TaPPR13*‐knockdown (KD) transgenic lines in Fielder (Figure S6a–f, Supporting Information) and treated the transgenic seedlings with 25% PEG6000 to induce water deficit conditions. After PEG6000 treatment, aerial biomass and root length were increased in the *TaPPR13*‐OE lines compared with the WT and *TaPPR13*‐KD lines (Figure S5g–i, Supporting Information), whereas there were no differences under normal conditions (Figure S6g–i, Supporting Information). These findings suggested that *TaPPR13* positively regulates drought stress tolerance in wheat.

**Figure 2:**
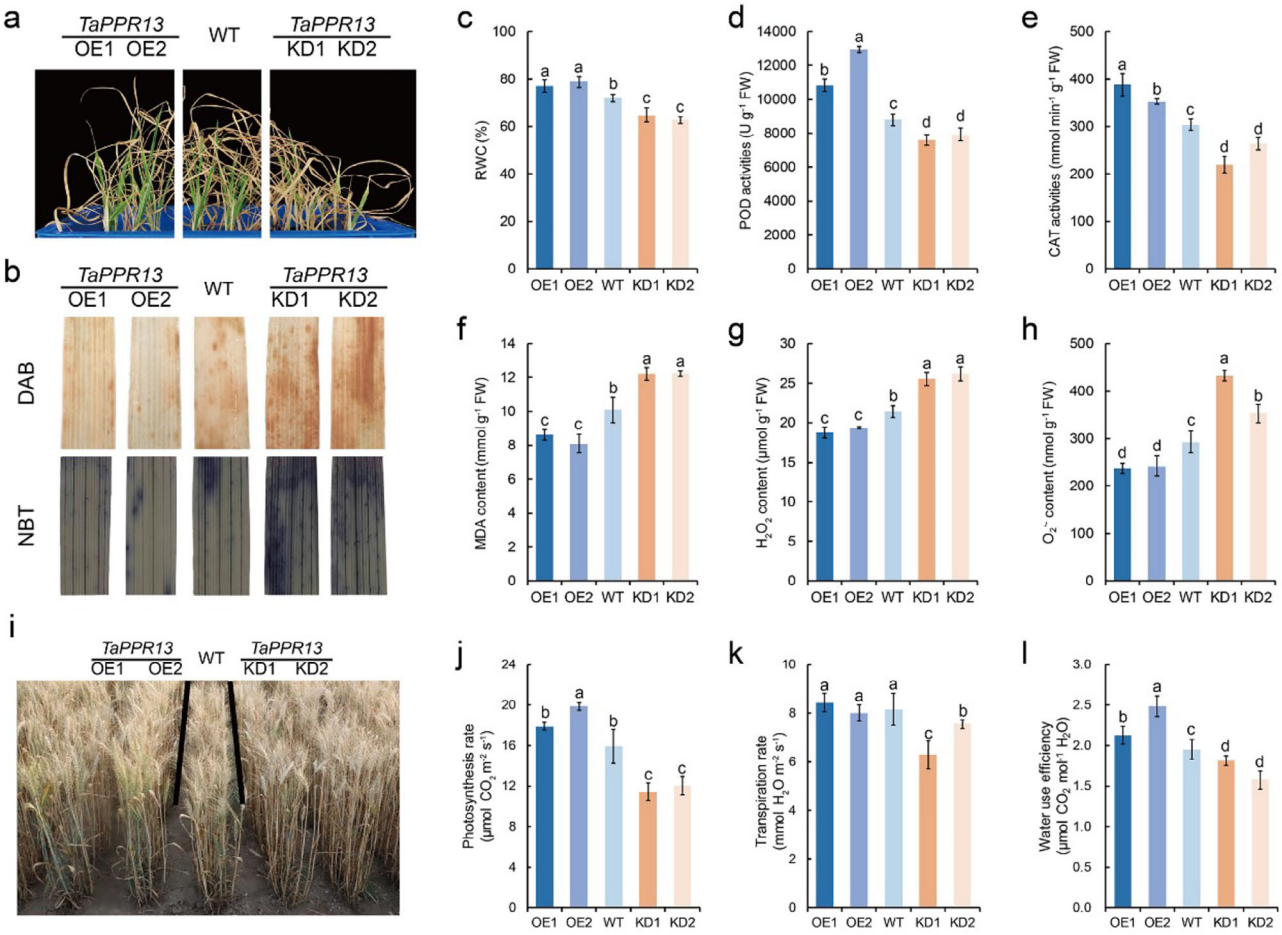
TaPPR13 positively enhances tolerance to drought stress. (a) Phenotypes of *TaPPR13*‐OE, WT, and *TaPPR13*‐KD plants under water deficit conditions. (b) DAB and NBT staining of *TaPPR13*‐OE, WT, and *TaPPR13*‐KD plants. (c) Relative water content (RWC) of plants grown under drought stress. Values are means ± SD (*p* < 0.05, *n* = 3, one‐way ANOVA, Tukey's HSD test). (d,e) POD and CAT activities of different plants under drought stress. Values are means ± SD (*p* < 0.05, *n* = 3, one‐way ANOVA, Tukey's HSD test). (f) MDA content of plants under drought stress. Values are means ± SD (*p* < 0.05, *n* = 3, one‐way ANOVA, Tukey's HSD test). (g,h) H_2_O_2_ and O_2_^•‐^ content of plants under drought stress. Values are means ± SD (*p* < 0.05, *n* = 3, one‐way ANOVA, Tukey's HSD test). (i) WT and transgenic wheat grown under drought stress conditions in the field. (j–l) Photosynthetic rate (j), transpiration rate, (k) and water use efficiency (l) of WT and transgenic wheat under drought stress conditions. Values are means ± SD (*p* < 0.05, *n* = 12, one‐way ANOVA, Tukey's HSD test).

To further investigate the function of TaPPR13 in drought tolerance, we planted the transgenic and WT plants in pots containing the same soil‐mix and simultaneously subjected them to water deficit stress (**Figure** **2**a). DAB and NBT staining demonstrated that *TaPPR13*‐OE plants exhibited reduced ROS production compared to WT and *TaPPR13*‐KD plants under drought stress conditions (Figure 2b). The *TaPPR13*‐KD plants exhibited lower relative water content (RWC) in their leaves compared with WT and *TaPPR13*‐OE plants (Figure 2c). Physiological indices revealed that the *TaPPR13*‐KD plants showed lower CAT and POD activities under drought stress conditions (Figure 2d,e), whereas there were no differences between WT and transgenic plants under normal conditions (Figure S7a–g, Supporting Information). *TaPPR13*‐OE plants accumulated lower levels of MDA, H_2_O_2_, and O_2_^•‐^ compared with WT and *TaPPR13*‐KD plants (Figure 2f–g). The transgenic plants were grown in standard field conditions to investigate the genetic effects of *TaPPR13* during flowering. Under water‐limited conditions, the photosynthesis rates, transpiration rates, and water use efficiency (WUE) in *TaPPR13*‐OE plants were greater than those of WT plants (Figure 2i–l).

### 2.3 Suppression of *TaPPR13* Resulted in Abnormal Chloroplast Formation Under Drought Stress

**Figure 3:**
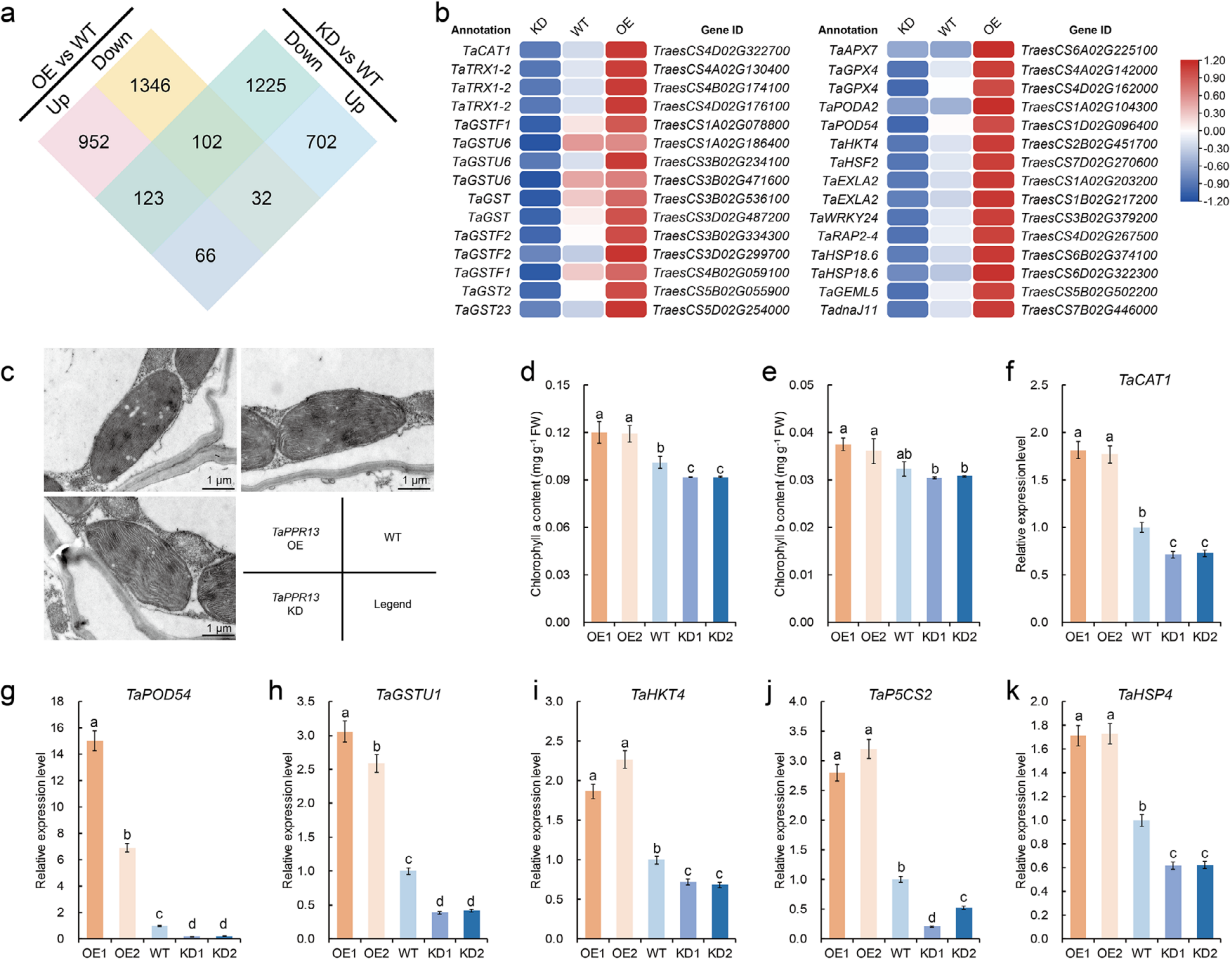
Effects of TaPPR13 on chloroplast formation and expression of abiotic‐stress responsive genes. (a) Numbers of differentially expressed genes (DEGs) between *TaPPR13*‐OE, WT, and *TaPPR13*‐KD plants subjected to drought stress. (b) Heat map representation of DEGs involved in ROS scavenging and abiotic‐stress response based on RNA‐seq analysis. (c) TEM images of chloroplast ultrastructure in WT and transgenic plants. (d,e) Chlorophyll a and b contents in *TaPPR13*‐OE, WT, and *TaPPR13*‐KD plants grown under drought stress. Values are means ± SD (*p* < 0.05, *n* = 3, one‐way ANOVA, Tukey's HSD test). (f–k) RT‐qPCR analysis of abiotic‐stress responsive genes identified from RNA‐seq data from plants grown under drought stress conditions. Values are means ± SD (*p* < 0.05, *n* = 4, one‐way ANOVA, Tukey's HSD test).

Transcriptome analysis carried out to elucidate the molecular mechanism of *TaPPR13*‐meditated tolerance to drought stress (Figure S8a, Supporting Information) indicated 1141 up‐regulated and 1,480 down‐regulated DEGs in *TaPPR13*‐OE plants, whereas there were 800 up‐regulated and 1450 down‐regulated DEGs in the *TaPPR13*‐KD line (**Figure** **3**a). GO enrichment analysis showed that the DEGs that responded to water deprivation, heat, and oxidative stress were significantly up‐regulated in the *TaPPR13*‐OE line under drought stress compared to WT plants (Figure S8b, Supporting Information). In addition, genes involved in stress response and ROS scavenging, including *TaCAT1*, *TaGSTs*, *TaPOD54*, *TaHSF2*, and *TaHSP18.6*, were up‐regulated in the *TaPPR13*‐OE plants, and down‐regulated in *TaPPR13*‐KD plants, compared with WT plants under drought stress conditions (Figure 3b). Notably, DEGs related to the chloroplast envelope were significantly up‐regulated in the *TaPPR13*‐OE line under drought stress (Figure S8b, Supporting Information). Since excessive ROS generation causes oxidative stress and damages chloroplast ultrastructure,^[^ ^7^, ^9^ ^]^ TEM was used to examine the chloroplast ultrastructure in WT and transgenic plants. Under drought stress, *TaPPR13*‐KD plants exhibited fractured thylakoid membranes (Figure 3c), whereas *TaPPR13*‐OE plants maintained higher chlorophyll a and b contents than WT plants (Figure 3d,e). Furthermore, RT‐qPCR analysis was conducted to investigate the variation in expression patterns of abiotic stress‐responsive genes influenced by the *TaPPR13* gene. The results showed that several key genes were up‐regulated following the increased expression of *TaPPR13* under drought stress. These included ROS scavenging‐related genes (*TaCAT1*, *TaPOD54*, and *TaGSTU1*), essential ion transporter gene *TaHKT4*, proline biosynthesis gene *TaP5CS2*, and molecular chaperone gene *TaHSP4*. (Figure 3f–k)

### 2.4 TaPPR13 Modulates ABA Sensitivity

**Figure 4:**
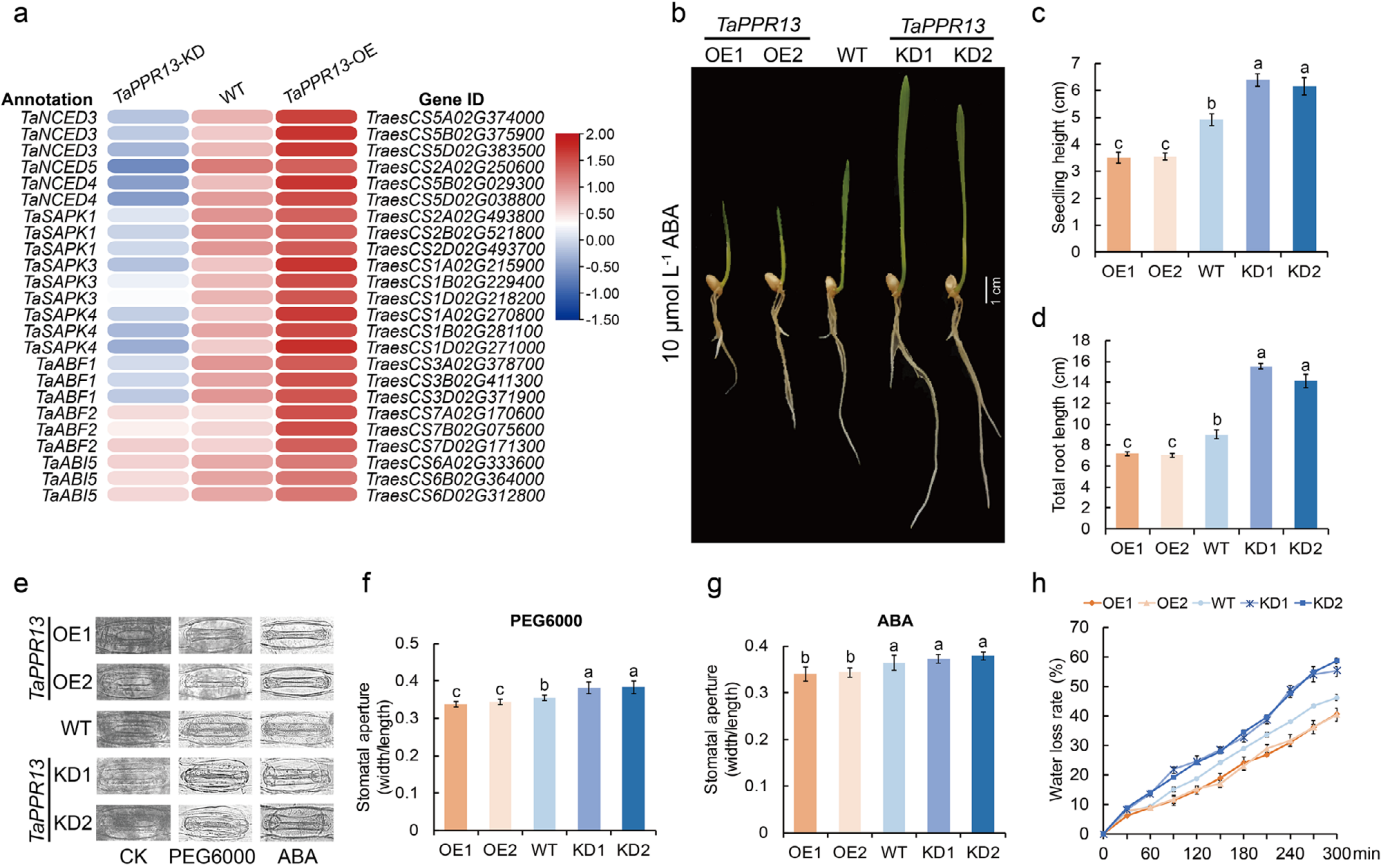
TaPPR13 increases ABA sensitivity and reduces water loss in wheat. (a) Heat map representation of DEGs involved in the ABA biosynthesis and signaling pathways, based on RNA‐seq analysis. (b) Phenotypes of *TaPPR13*‐OE, WT, and *TaPPR13*‐KD seedlings after ABA treatment. (c,d) Seedling height (c) and total root length (d) of *TaPPR13*‐OE, WT, and *TaPPR13*‐KD seedlings. Values are means ± SD (*p* < 0.05, *n* = 8, one‐way ANOVA, Tukey's HSD test). (e–g) Stomatal aperture of *TaPPR13*‐OE, WT, and *TaPPR13*‐KD plant leaves in response to drought stress and ABA treatment. Values are means ± SD (*p* < 0.05, *n* = 15, one‐way ANOVA, Tukey's HSD test). (h) Water loss rate of *TaPPR13*‐OE, WT, and *TaPPR13*‐KD plant. Values are means ± SD (*p* < 0.05, *n* = 3, one‐way ANOVA, Tukey's HSD test).

The ABA transduction pathway plays a role in response to abiotic stress.^[^ ^38^, ^39^, ^40^ ^]^ Transcriptome analysis showed that DEGs known to responded to the ABA signal pathway were significantly enriched in *TaPPR13*‐OE plants under drought stress conditions (Figure S8b, Supporting Information). Additionally, ABA biosynthesis genes (*TaNCEDs*), SnRK2 genes (*TaSAPKs*), and key TF genes in the ABA signal pathway (*TaABFs* and *TaABIs*) were significantly induced in *TaPPR13*‐OE plants compared to WT plants (**Figure** **4**a). Furthermore, comparison of ABA sensitivity between *TaPPR13*‐OE, WT and *TaPPR13*‐KD plants (Figure 4b) showed that seedling height and root length of *TaPPR13*‐OE plants were significantly more inhibited by ABA than those of WT and *TaPPR13*‐KD plants (Figure 4c,d). ABA and PEG6000 applied to the leaves indicated increased stomatal closure in *TaPPR13*‐OE plants compared with WT and *TaPPR13*‐KD plants (Figure 4e–g), and rate of water loss in *TaPPR13*‐OE lines was reduced relative to WT plants (Figure 4h). These results indicated that TaPPR13 improved resistance to drought stress by enhancing ABA sensitivity and mediating the ABA signaling pathway.

### 2.5 TaPPR13 Interacts with TaAOR1 to Enhance Detoxification Processes

**Figure 5:**
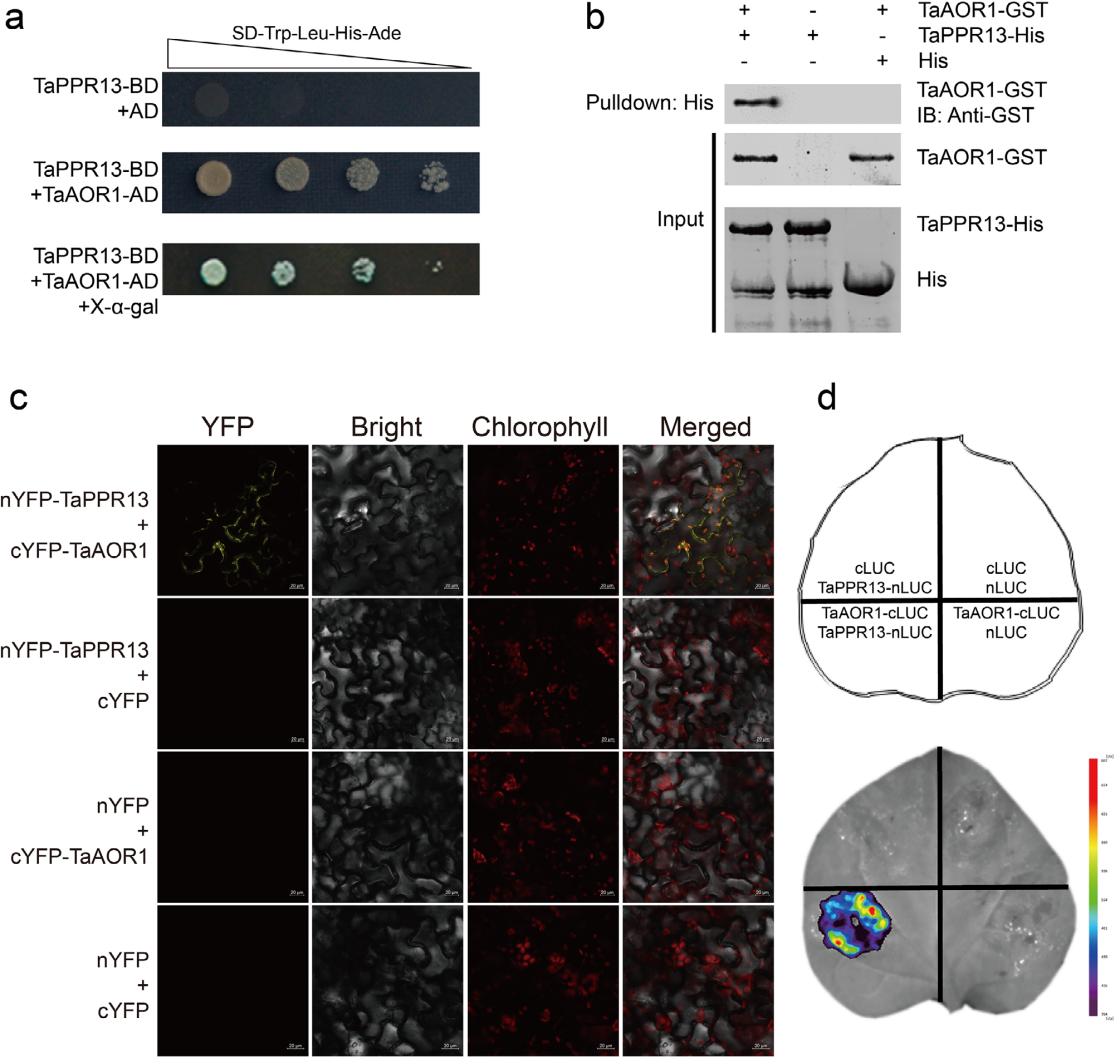
Interaction between TaPPR13 and TaAOR1. (a) Verification of protein interaction of TaPPR13 and TaAOR1 by yeast two‐hybrid assay. (b) Pull‐down assay demonstrating that TaPPR13 interacts with TaAOR1 in vitro. (c,d) BiFC and LCI assay revealing the interaction between TaPPR13 and TaAOR1 in tobacco leaves.

Y2H assays were carried out using TaPPR13 protein as bait to identify other components of TaPPR13‐medited drought stress response. TaAOR1, a nuclear‐encoded chloroplast localized alkenal/one oxidoreductase, was identified as the interacting protein of TaPPR13 (**Figure** **5**a; Figure S9a, Supporting Information). Through pull‐down assays, we confirmed that the TaPPR13‐His protein directly interacts with TaAOR1‐GST in vitro (Figure 5b). Interaction between the TaPPR13 and TaAOR1 proteins was also confirmed by bimolecular fluorescence complementation (Figure 5c) and luciferase complementation (Figure 5d) assays.

**Figure 6:**
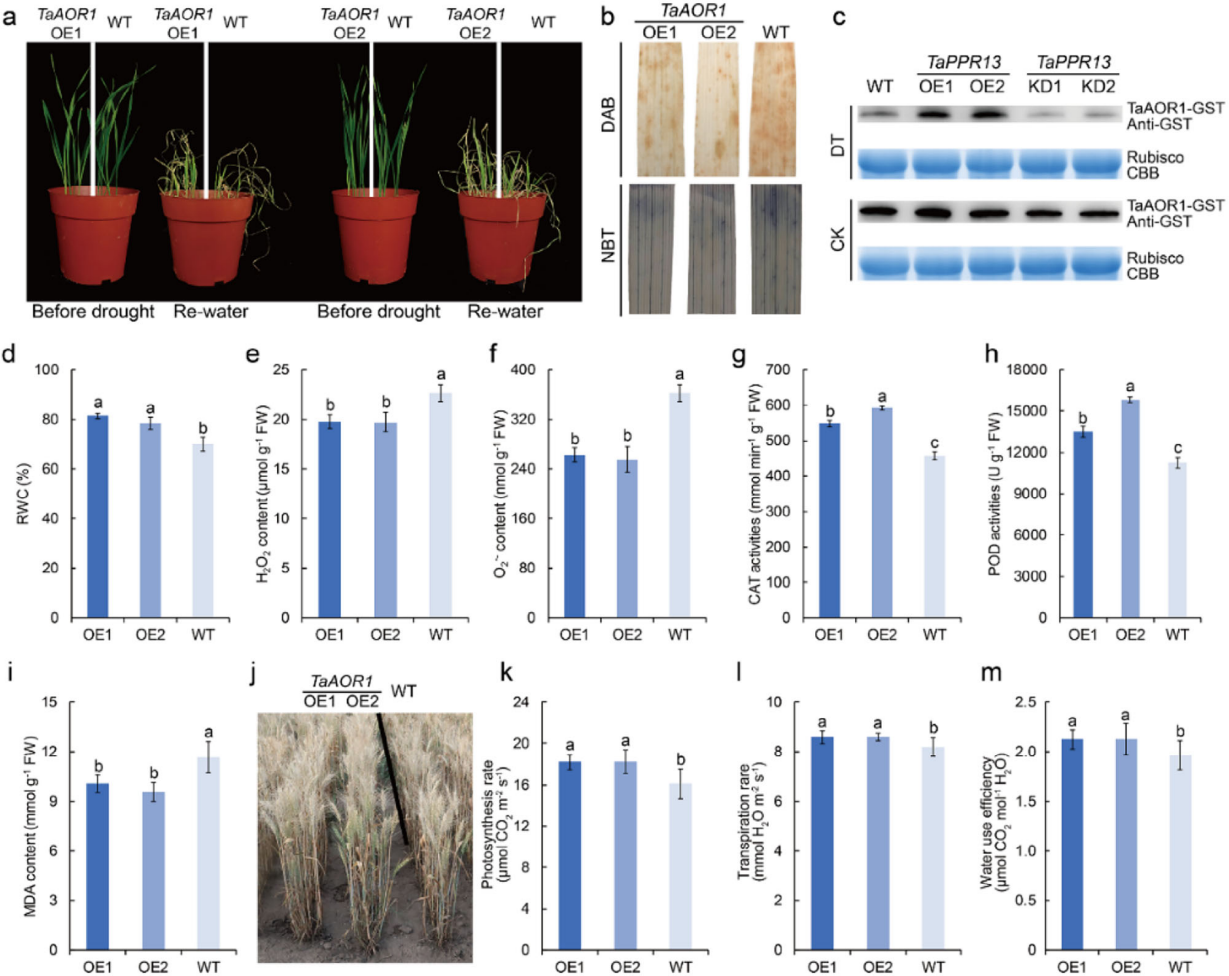
TaAOR1 enhances drought stress tolerance in wheat. (a) Phenotypes of *TaAOR1*‐OE and WT plants under water deficit conditions. (b) DAB and NBT staining. (c) TaPPR13 stabilizes TaAOR1 under drought stress. In vitro, degradation assays the degradation of TaAOR1‐GST in leaf‐protein extracts of *TaPPR13*‐OE, WT, and *TaPPR13*‐KD plants. Ten‐day‐old plants of *TaPPR13*‐OE, WT, and *TaPPR13*‐KD were treated with or without 20% PEG6000 for 2 days. Immunoblots were probed with anti‐GST antibody. Rubisco was used as a loading control. (d) Relative water content (RWC) of *TaAOR1*‐OE and WT plants under drought stress. Values are means ± SD (*p* < 0.05, *n* = 3, one‐way ANOVA, Tukey's HSD test). (e,f) Measurement of H_2_O_2_ and O_2_^•‐^ levels in different plants under drought stress. Values are means ± SD (*p* < 0.05, *n* = 3, one‐way ANOVA, Tukey's HSD test). (g,h) CAT and POD activities in plants under drought stress. (i) MDA content of different lines under drought stress. Values are means ± SD (*p* < 0.05, *n* = 3, one‐way ANOVA, Tukey's HSD test). (j) Phenotypes of WT and transgenic wheat under drought stress condition in the field. (k–m) Photosynthetic rate, transpiration rate, and water use efficiency of WT and transgenic wheat grown under drought stress conditions. Values are means ± SD (*p* < 0.05, *n* = 12, one‐way ANOVA, Tukey's HSD test).

RT‐qPCR analysis to investigate whether *TaAOR1* responded to drought stress showed that the expression level of *TaAOR1* was markedly induced under drought stress (Figure S9b,c, Supporting Information), and alignment analysis showed that the TaAOR1 protein is closely related to AtAOR1 (Figure S9d, Supporting Information). Previous research showed that chloroplastic AtAOR1 contributes to the detoxification under oxidative stress.^[^ ^41^ ^]^ To verify the function of TaAOR1 on drought stress tolerance, we generated *TaAOR1*‐OE transgenic lines in Fielder (Figure S9e,f, Supporting Information). NBT and DAB staining indicated that *TaAOR1*‐OE plants exhibited reduced ROS production compared to WT plants under water deficit stress (Figure 6b). Furthermore, in vitro degradation assays were performed to check the abundance of TaAOR1‐GST protein in leaf‐protein extracts of *TaPPR13*‐OE, WT, and *TaPPR13*‐KD plants. Our results showed that TaAOR1 abundance was decreased significantly in *TaPPR13*‐KD plants under drought stress, whereas *TaPPR13*‐OE plants could stabilize TaAOR1 under drought stress (Figure 6c).

**Figure 7:**
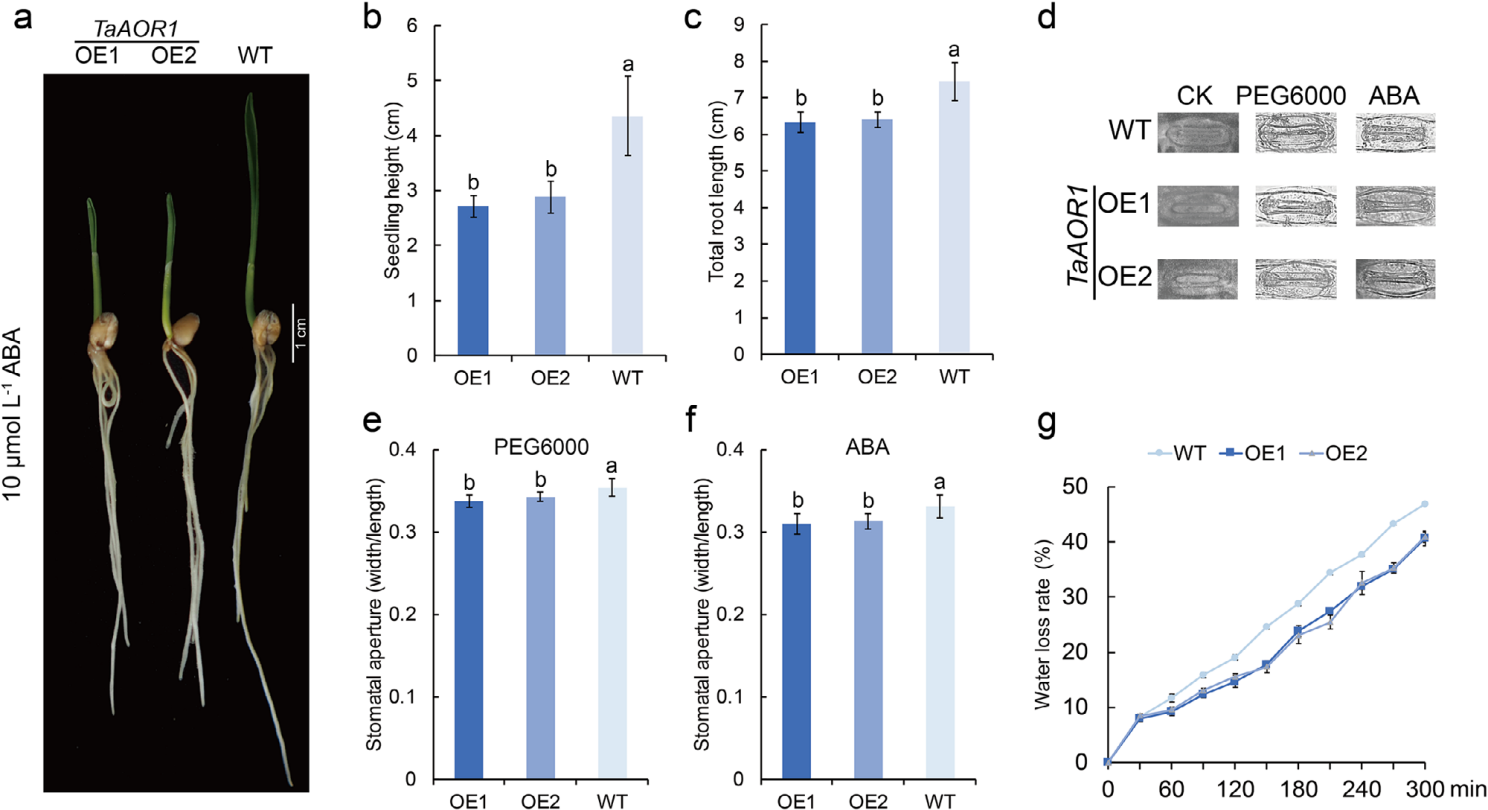
TaAOR1 increases ABA sensitivity and reduces water loss in wheat. (a) Phenotypes of *TaAOR1*‐OE and WT plants after ABA treatment; Bar = 1 cm. (b,c) Seedling height (b) and total root length (c) of *TaAOR1*‐OE and WT seedlings. Values are means ± SD (*p* < 0.05, *n* = 8, one‐way ANOVA, Tukey's HSD test). (d–f) Stomatal apertures in leaves of *TaAOR1*‐OE and WT plant following drought stress and ABA treatments. Values are means ± SD (*p* < 0.05, *n* = 15, one‐way ANOVA, Tukey's HSD test). (g) Water loss rate of *TaAOR1*‐OE and WT plant. Values are means ± SD (*p* < 0.05, *n* = 3, one‐way ANOVA, Tukey's HSD test).

Under drought stress condition, *TaAOR1*‐OE plants also exhibited higher RWC in leaves compared with WT plants (Figure 6d), whereas the WT plants accumulated more O_2_^•‐^ and H_2_O_2_ than *TaAOR1*‐OE lines (Figure 6e,f). In contrast to the WT plants, *TaAOR1*‐OE lines showed higher CAT and POD activities, but reduced accumulation of MDA (Figure 6g–i). Photosynthesis and transpiration rates, and WUE of *TaAOR1*‐OE plants subjected to water deficit at flowering stage were higher than those of WT controls (Figure 6j–m). Furthermore, TEM showed that WT plants had abnormal chloroplasts with fractured thylakoid membranes (Figure S10a, Supporting Information), whereas the *TaAOR1*‐OE lines maintained thylakoid structure and had higher chlorophyll a and b contents than the WT (Figure S10b,c, Supporting Information). RT‐qPCR analysis showed that the expression of abiotic stress‐responsive genes was more highly induced in *TaAOR1*‐OE lines than in WT plants when exposed to drought stress (Figure S10d–i, Supporting Information). Additionally, ABA sensitivity experiments demonstrated that seedling height and root length in *TaAOR1*‐OE lines were reduced relative to WT following application (**Figure** **7**a–c), and both ABA and PEG6000 increased stomatal closure of *TaAOR1*‐OE lines compared with WT plants (Figure 7d–f), whereas the rate of water loss in *TaAOR1*‐OE lines was reduced compared with WT plants (Figure 7g).

### 2.6 TaPPR13 Interacts with TaSIG5 and Regulate Chloroplast Gene Expression

**Figure 8:**
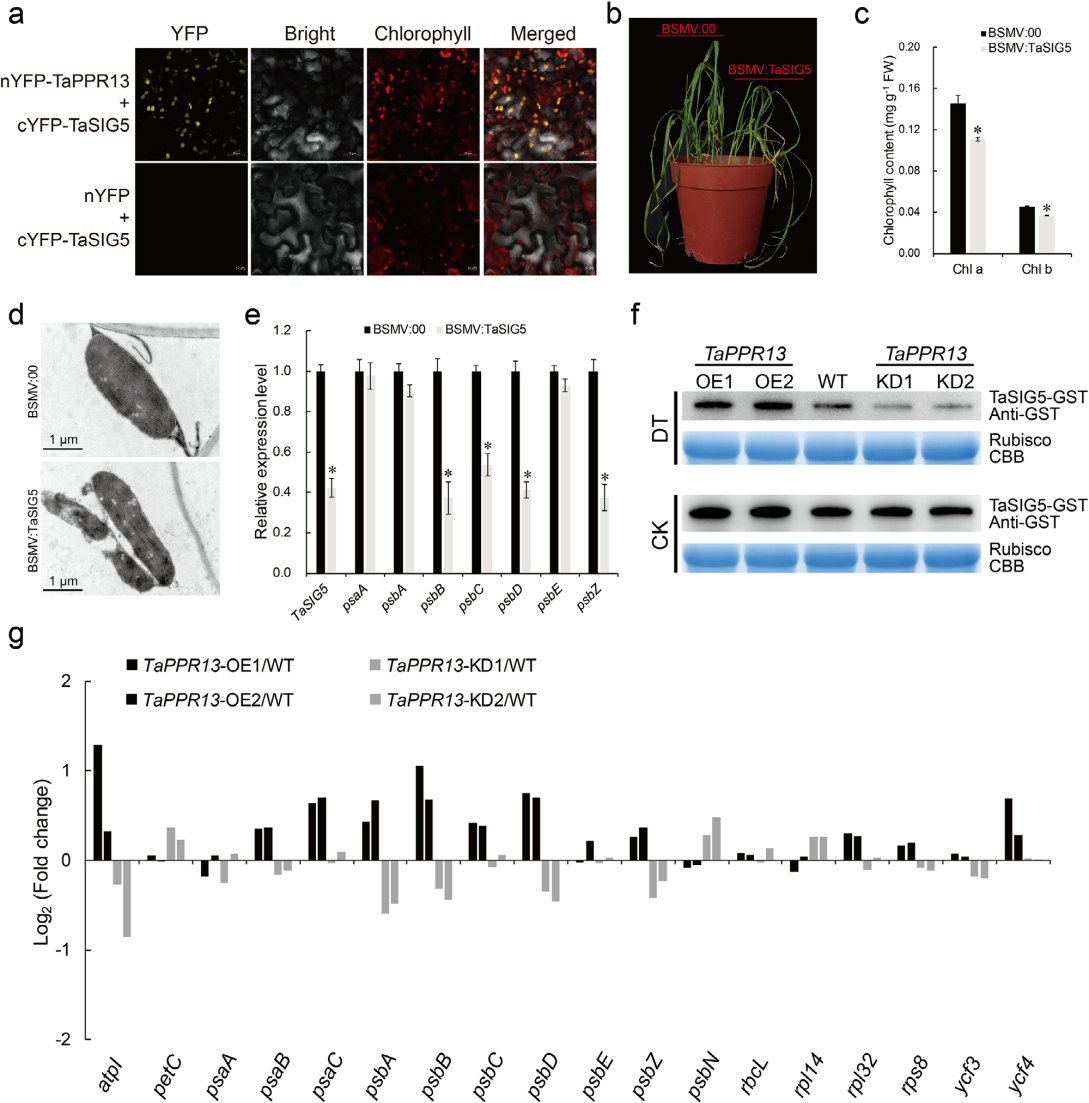
TaPPR13 interacts with TaSIG5 and modifies chloroplast gene expression. (a) BiFC assay demonstrates that TaPPR13 interacts with TaAOR1 in tobacco leaves. (b) Phenotypes of *TaSIG5* knockdown plants grown under drought stress conditions. (c) Chlorophyll contents of *TaSIG5* knockdown and empty vector control plants under drought stress. (d) TEM images of chloroplast ultrastructure in BSMV:00 and BSMV:TaSIG5 plants. (e) RT‐qPCR analysis of expression patterns of PEP‐dependent genes in *TaSIG5* knockdown and empty vector control plants grown under water deficit conditions. Values are means ± SD (*p* < 0.05, *n* = 4, Student's *t*‐test). (f) In vitro, degradation assays the degradation of TaSIG5‐GST in leaf‐protein extracts of *TaPPR13*‐OE, WT, and *TaPPR13*‐KD plants. Ten‐day‐old plants of *TaPPR13*‐OE, WT, and *TaPPR13*‐KD were treated with or without 20% PEG6000 for 2 days. Immunoblots were probed with anti‐GST antibody. Rubisco was used as a loading control. (g) RT‐qPCR analysis of expression patterns of chloroplast‐encoded genes in *TaPPR13*‐OE, WT, and *TaPPR13*‐KD plants grown under water deficit conditions; bars indicate the log_2_ ratio of expression levels in transgenic plants compared with the WT.

TaSIG5, a chloroplast localized sigma factor, was identified as the interacting protein of TaPPR13 by Y2H assay (Figure S11a,b, Supporting Information). BiFC assays further showed that TaPPR13 physically interacts with TaSIG5 within the chloroplasts (**Figure** **8**a). Alignment analysis showed that the TaSIG5 protein is closely related to OsSIG5 and AtSIG5 (Figure S11c, Supporting Information). Previous research showed that SIG5 functioned as a multiple‐stress responsive sigma factor that protected plants from stress by enhancing repair mechanisms in the PSII reaction center.^[^ ^42^, ^43^ ^]^ RT‐qPCR analysis to investigate whether *TaSIG5* responds to drought stress indicated that the expression level of *TaSIG5* was markedly induced under drought stress (Figure S11d,e, Supporting Information). The VIGS assay in cultivar Pubingzi300 showed that *TaSIG5*‐silenced plants had severely wilted leaves and lower chlorophyll content compared with the control plants under drought stress (Figure 8b,c; Figure S11f, Supporting Information), and TEM showed that *TaSIG5*‐silenced plants had abnormal chloroplasts with fractured thylakoid membranes (Figure 8d). Expression of chloroplast genes in *TaSIG5*‐silenced plants measured by RT‐qPCR showed that plastid‐encoded polymerase (PEP)‐dependent chloroplast genes (such as *psbB*, *psbC*, *psbD*, and *psbZ*) were markedly suppressed in *TaSIG5*‐silenced plants (Figure 8e). In vitro degradation assays were performed to check the abundance of TaSIG5‐GST protein in leaf‐protein extracts of *TaPPR13*‐OE, WT, and *TaPPR13*‐KD plants. The results showed that TaSIG5 abundance was decreased significantly in *TaPPR13*‐KD plants under drought stress, whereas *TaPPR13*‐OE plants stabilized TaSIG5 under drought stress (Figure 8f). Furthermore, RT‐qPCR analysis showed that the PEP‐dependent chloroplast genes were markedly suppressed in *TaPPR13*‐KD plants, but were upregulated in *TaPPR13*‐OE lines compared with control plants grown under drought stress conditions (Figure 8g).

### 2.7 TaPPR13 and TaAOR1 Enhanced Yield Performance

**Figure 9:**
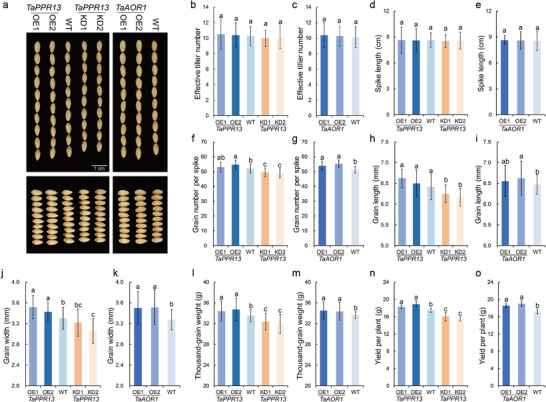
TaPPR13 and TaAOR1 enhanced yield performance. (a) Grain length and width phenotypes of transgenic wheat and WT plants. (b–e) Statistical data for effective tiller number (b,c) and spike length (d,e) of WT and transgenic wheat plants grown under drought stress conditions in the field. Values are means ± SD (*p* < 0.05, *n* = 8, one‐way ANOVA, Tukey's HSD test). (f–o) Statistical data for grain number per spike (f,g), grain length (h,i), grain width (j,k), thousand kernel weight (l,m), and yield per plant (n,o) of WT and transgenic wheat plants grown under drought stress conditions in the field. Values are means ± SD from three independent experiments (*n* > 30, *p* < 0.05, one‐way ANOVA, Tukey's HSD test).

Transgenic plants were grown in standard field conditions to investigate the genetic effects of *TaPPR13* and *TaAOR1* during the reproductive stages. Under well‐watered conditions, no difference was observed between the WT and transgenic plants in terms of effective tiller number, spike length, grain size, and grain weight (Figure S12 and Table S2, Supporting Information). Under drought stress, no difference was observed between the WT and transgenic plants for effective tiller number and spike length (**Figure** **9**b–e and Table S2, Supporting Information). However, *TaPPR13*‐OE and *TaAOR1*‐OE plants produced higher grain numbers and grain kernel weights, whereas *TaPPR13*‐KD plants showed smaller grain size and produced lower grain numbers and thousand‐grain weight than WT plants (Figure 9f–m and Table S2, Supporting Information). Moreover, the *TaPPR13*‐OE and *TaAOR1*‐OE lines displayed 4.5–8.2% and 7.9–10.7% higher grain yield per plant, respectively, than WT plants under drought stress (Figure 9n,o).

## 3 Discussion

**Figure 10:**
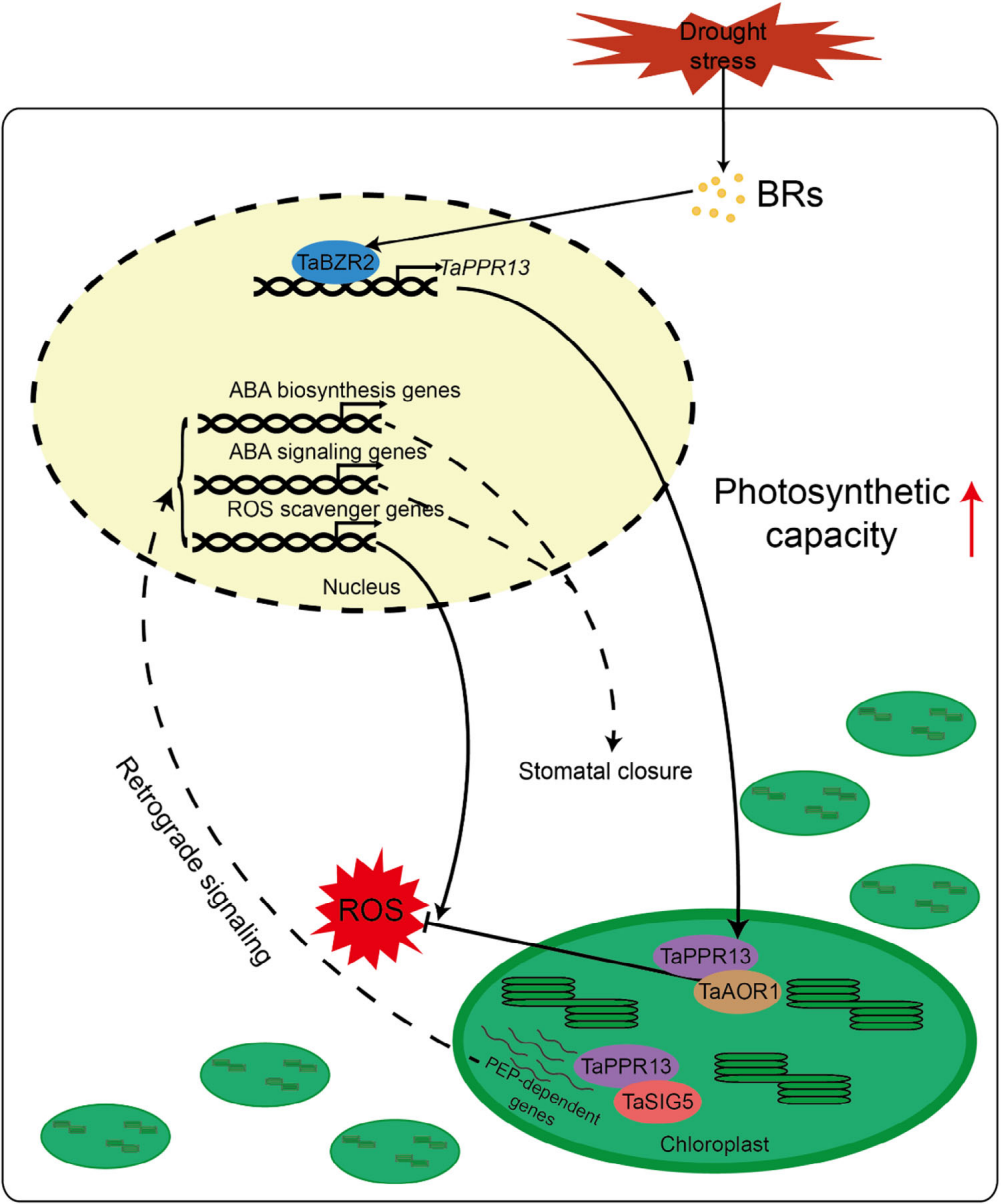
Model of TaPPR13‐mediated drought response in wheat plants. TaPPR13 functions as a positive regulator downstream of TaBZR2, further strengthening drought tolerance by interacting with TaAOR1 and TaSIG5 to improve the antioxidant defense and regulate chloroplast gene expression.

BES/BZRs, as core TFs of the BR signaling pathway, have been implicated in plant responses to abiotic stresses, including heat,^[^ ^44^, ^45^ ^]^ drought,^[^ ^35^ ^]^ freezing,^[^ ^46^ ^]^ and salinity.^[^ ^47^, ^48^ ^]^ In this study, we determined that TaBZR2 was significantly associated with drought tolerance, and the WUE and agronomic traits in *TaBZR2*‐OE plants were better than those of WT plants (Figure 1). Our previous study showed that TaBZR2 enhanced drought stress tolerance in wheat by activating the *TaGST1* gene and scavenging of O_2_^•‐^.^[^ ^35^ ^]^ Previous studies showed that TaBZR1 interacted with TaHAG1 (histone acetyltransferase) to induce the expression of TaSAMT1 (methyltransferase) and modulated the salicylic acid (SA) pathway during freezing stress,^[^ ^46^ ^]^ while Yang et al.^[^ ^49^ ^]^ elucidated that TaBZR1 conferred salinity stress tolerance by activating the expression of ABA biosynthesis and ROS scavenging genes. Previous studies reported that early short‐term BR signal activation was linked to ABA‐mediated abiotic stress tolerance,^[^ ^50^, ^51^, ^52^ ^]^ and BZR1 enhanced tomato chilling tolerance via ABA biosynthesis.^[^ ^53^ ^]^ In this study, TaBZR2 positively activated expression of *TaPPR13* by binding to its E‐box *cis*‐elements, and overexpression of *TaPPR13* improved drought stress tolerance by retrograde signaling to alter nuclear‐encoded ABA biosynthesis and signal pathway genes (**Figure** **10**). Furthermore, *TaPPR13* was identified as a key factor influencing the response of wheat to ABA signaling. Sensitivity of *TaPPR13*‐OE plants to ABA is significantly enhanced, whereas the sensitivity of *TaPPR13*‐KD transformed wheat lines to ABA is markedly reduced. These results suggested that TaBZR2 might function in synergistic interplay of ABA and BR signals in regulating adaptation to abiotic stress.

Our present results demonstrated that overexpression of the *TaPPR13* gene enhanced drought tolerance in wheat by modulating chloroplastic ROS homeostasis and maintaining chloroplast structure (Figure 3). Additionally, the expression of ABA biosynthesis and signaling pathway genes was significantly downregulated in *TaPPR13*‐KD transgenic wheat plants under drought stress (Figure 4). Recent studies found that PPR proteins are involved in environmental response.^[^ ^54^, ^55^, ^56^, ^57^ ^]^ The PPR protein SOAR1 is positively involved in response to drought and salt stress by influencing ABA signaling.^[^ ^32^, ^33^ ^]^ Loss function of PPR protein SOP10 suppressed O_2_^•‐^ accumulation and enhanced cold stress tolerance in rice.^[^ ^58^ ^]^ Knockdown of PPR protein gene *PPS1* in rice effected the ABA and ROS signaling pathways and exerted hypersensitivity to abiotic stress.^[^ ^21^ ^]^ The Arabidopsis PPR protein POCO1 functions in ABA‐dependent drought signal transduction.^[^ ^59^ ^]^ These findings suggest that PPR proteins function in a highly complicated mechanism to control the ROS homeostasis and play a role in ABA signaling and stress response.^[^ ^30^, ^32^ ^]^ Under adverse circumstances, chloroplasts overproduce ROS causing oxidative damage,^[^ ^7^, ^9^, ^60^ ^]^ and increased ROS levels in plants lead to the accumulation of reactive carbonyl species (RCS), which can mediate ROS signals to proteins in response to oxidative stress.^[^ ^61^, ^62^ ^]^ Plants have developed a highly efficient antioxidant system to maintain a balance between production and removal of ROS and RCS within individual cellular compartments.^[^ ^61^, ^63^ ^]^ Previous research showed that overexpression of ROS scavenging enzymes and RCS scavenger enzymes is often associated with increased plant stress tolerance.^[^ ^41^, ^62^, ^64^, ^65^ ^]^ Overexpression of the aldehyde dehydrogenase (ALDH) *ScALDH21* gene from *Syntrichia caninervis* in tobacco and cotton resulted in higher activities of ROS scavenging enzymes, stronger photosynthetic capacity, and higher yield under drought stress.^[^ ^66^, ^67^ ^]^ Wheat TaWD40‐4B.1^C^ interacted with canonical catalases to avoid ROS over‐accumulation and enhanced grain yield under water‐withheld conditions.^[^ ^68^ ^]^ In the present study, TaAOR1 interacts with TaPPR13, and *TaPPR13*‐OE stabilized TaAOR1 to reduce the accumulation of MDA and ROS under drought stress conditions (Figure 6). These results showed that TaPPR13 and TaAOR1 positively enhance the antioxidant defense system to protect chloroplast structure. Moreover, enhanced *TaPPR13* and *TaAOR1* expression improved photosynthetic efficiency to enhance productivity under water deficit conditions (Figure 9). Similar benefits of antioxidant defense in increasing yield have been reported in genetically modified drought‐tolerant rice,^[^ ^69^ ^]^ wheat,^[^ ^68^ ^]^ maize,^[^ ^70^ ^]^ and soybean^[^ ^71^ ^]^ genotypes.

ROS‐mediated retrograde signaling is a communication mechanism that allows organelles to signal to the nucleus to regulate gene expression,^[^ ^72^, ^73^, ^74^, ^75^ ^]^ and is an important aspect of plant response to abiotic stress.^[^ ^73^, ^76^, ^77^ ^]^ Arabidopsis PPR40 protein responds to oxidative stress by altering ROS homeostasis and stress‐responsive gene expression.^[^ ^78^ ^]^ Mitochondrion‐localized PPR ABO5 proteins regulate the expression of stress‐inducible genes (such as *ABF2* and *RD29A*) impacting ABA signaling.^[^ ^79^ ^]^ Expression of *ABI5* was significantly downregulated in plant with *ppr96* and *poco1* mutant alleles.^[^ ^59^, ^80^ ^]^ ABF and ABI5 transcription factors serve as master regulators of ABA signaling in response to drought stress.^[^ ^38^, ^81^ ^]^ Under drought stress conditions, rapid ABA and ROS accumulation leads to stomatal closure to reduce water loss.^[^ ^82^, ^83^, ^84^, ^85^ ^]^ Stomatal transpiration accounts for ≈95% of total water loss in plants,^[^ ^86^, ^87^ ^]^ and reduction of water loss through stomates is an effective adaptation strategy in improving drought stress tolerance.^[^ ^88^, ^89^, ^90^, ^91^ ^]^ Maize ZmCPK35/37 regulate stomatal closure to retain more water in leaves and enhance maize yield under drought stress.^[^ ^92^ ^]^ OsASR5 and TaNAC48 confer drought stress tolerance through a stomatal closure pathway by increased endogenous ABA biosynthesis.^[^ ^90^, ^93^ ^]^ Mutant of lysine deacetylase TaSRT1 displayed lower transpiration levels, less water loss, higher net photosynthetic rate, and water use efficiency under drought stress conditions.^[^ ^94^ ^]^ In this study, the expression levels of genes involved in ABA biosynthesis and signaling pathways, including *TaNCED3*, *TaNCED4*, *TaABF1/2*, and *TaABI5*, were significantly upregulated in *TaPPR13*‐OE lines and downregulated in *TaPPR13*‐KD plants (Figure 4). Moreover, overexpression of the *TaPPR13* and *TaAOR1* genes increased stomatal closure to mitigate leaf water loss (Figures 4 and 7). Furthermore, our results showed that TaPPR13 functions in regulation of chloroplast gene expression by interacting with TaSIG5 (Figure 8), leading to enhanced rates of photosynthesis and transpiration, and increased photosynthetic rate and water use efficiency in *TaPPR13*‐OE plants (Figure 2). Previous studies showed that sigma factors (SIGs) function in retrograde signaling to control nuclear gene expression.^[^ ^42^, ^43^, ^95^ ^]^ For instance, SIG5 functions in chloroplast transcriptional response to abiotic stress,^[^ ^43^, ^96^ ^]^ and SIG2 and SIG6 have partially redundant roles in retrograde signaling to regulate nuclear gene expression.^[^ ^95^ ^]^

In conclusion, we identified a PPR protein family gene, *TaPPR13*, in wheat that is activated by TaBZR2 to enhance drought stress tolerance and photosynthetic capacity. It achieves this by interacting with TaAOR1 and TaSIG5 to improve antioxidant defense and regulate chloroplast gene expression (Figure 10).

## 4 Experimental Section

### Plant Materials and Treatments

All plants were grown in an LEDR‐1000 plant growth chamber (Yanghui, Ningbo, China) with 70% relative humidity and 25/20 °C day/night temperatures. For analysis of gene expression patterns, 7‐day‐old hydroponically grown seedlings were subjected to treatments with PEG6000, ABA, and BR. Leaves for RNA isolation were harvested at different time points (0, 2, 4, 8, 12, and 24 h) and immediately frozen in liquid nitrogen, and stored at −80 °C. The 3‐day‐old seedlings were exposed to 25% PEG6000 for 5 days to simulate water stress treatment. For the drought stress phenotyping, wheat seedlings were grown in the pots containing the same soil mix. For drought tests under field conditions, wheat lines were grown in an experimental field at Beijing (40°13′52″ N, 116°33′52″ E) as previously described.^[^ ^6^ ^]^ The plant leaf photosynthesis rates (PS) and transpiration rates (TR) were measured using a LI‐COR LI6800 portable photosynthesis system as described previously.^[^ ^4^ ^]^ Water use efficiency (WUE) was calculated as the ratio of PS to TR according to the method outlined by Wang et al.^[^ ^97^ ^]^ Agronomic traits, including effective tiller number per plant, spike length, grain number per spike, and grain yield per plant, were measured following harvest. Grain length, grain width, and grain kernel weight were analyzed using the SC‐G automatic seeds test system (Wanshen Ltd., Hangzhou, China). Stress‐tolerant wheat varieties (including wheat cv. Pubingzi300, Pinyu8012, Xinong877, Zhengmai1860, Jimai60, Jinhe991 and Ningmai58) were selected for gene cloning. No variation in the coding sequences (CDS) of *TaPPR13* was detected.

### Physiological Measurement

For physiological characteristics, wheat leaves were harvested after treatment according to methods described previously.^[^ ^6^ ^]^ The relative water content (RWC) of leaves was calculated using the published formula.^[^ ^40^ ^]^ Chlorophyll a, chlorophyll b, malondialdehyde (MDA), hydrogen peroxide (H_2_O_2_), and superoxide radical (O_2_^•‐^) contents, along with catalase (CAT) and peroxidase (POD) activities were determined using physiological assay kits from Solarbio (Beijing). For 3,3′‐diaminobenzidine (DAB) and nitro blue tetrazolium (NBT) staining, the DAB (1 mg mL^−1^, pH3.8, Coolaber) and NBT (0.5 mg mL^−1^, pH7.8, Coolaber) solution were prepared according to the manufacturer's instructions.

### RNA Isolation, Transcriptome Analysis and RT‐qPCR

Total RNA for transcriptome analysis was isolated using an RNA Easy Fast Plant Tissue Kit (Tiangen), and RNA‐seq was carried out by Tiangen Biotech Co. Ltd (Beijing). Differentially expressed genes (DEGs) were identified by DESeq2 with |log2(FoldChange)| > 1 and *p*‐value < 0.05, and the raw reads were submitted to the NCBI Sequence Read Archive (SRA) under Bioproject ID: PRJNA1056048. First‐strand cDNA for RT‐qPCR analysis was synthesized using a FastKing RT Kit with gDNase (Tiangen) according to the manufacturer's instructions. RT‐qPCR conducted on the TGreat Real qPCR system (OSE‐R96) using Talent qPCR PreMix (SYBR Green) followed the manufacturer's protocol. All primers used in this study are listed in Table S3 (Supporting Information), and the expression levels were calculated using the 2^‐ΔΔCT^ method.^[^ ^98^ ^]^

### ABA Sensitivity Assays

Germinating seedlings at two days postwetting were treated with Hoagland's solution containing 10 µmol L^−1^ ABA for 5 days for determination seedling height and root length. Seedling height was measured using a ruler, while total root length was measured using a WinRHIZO root scanning equipment and imaging system (Regent Instruments Inc., Canada) coupled with an Epson Expression 10000XL Pro scanner. The water loss rate was measured according to published method.^[^ ^93^, ^99^ ^]^

### Stomatal Measurement

Leaves from 15‐day‐old *TaPPR13* and *TaAOR1* transgenic and WT seedlings were incubated in MES solution buffer (10 mmol L^−1^ MES, 10 mmol L^−1^ KCl, 50 µmol L^−1^ CaCl_2_, pH 6.15) for 2 h to induce stomatal opening.^[^ ^100^ ^]^ After incubation, the leaves were transferred to a MES buffer solution containing 10% PEG6000 or 10 µmol L^−1^ ABA for an additional 2 h. Stomatal observations were made using an OLYMPUS BX51 microscope (Tokyo, Japan) and stomatal apertures were measured as previously described.^[^ ^3^ ^]^

### Transmission Electron Microscope (TEM) Analysis

Leaves from WT, *TaPPR13*, and *TaAOR1* transgenic plants, BSMV:00 and BSMV:TaSIG5 for chloroplast ultrastructure analysis were collected and fixed using electron microscope fixative solution (Servicebio, Wuhan). The chloroplast ultrastructure was viewed using a Hitachi TEM system (Tokyo).

### Yeast Hybrid Assay

To identify transcription factors regulating *TaPPR13* expression a 2‐kb promoter of *TaPPR13* was cloned into the pHis2 vector and a Y1H screen assay was carried out using a Y187‐pHis2 Yeast One‐Hybrid Library Screening kit (Coolaber) following the manufacturer's protocol. For Y2H assays, the coding sequence of *TaPPR13* was cloned into the pGBKT7 vector, and the full‐length sequences of *TaAOR1* and *TaSIG5* were cloned into the pGADT7 vector. The Y2H assay was performed using an AH109‐GAL4 Yeast Two‐Hybrid interaction kit (Coolaber) following the manufacturer's protocol.

### Subcellular Localization, BiFC, LCI, and LUC Assay

The 35S::TaPPR13‐GFP, 35S::TaAOR1‐GFP, and 35S::TaSIG5‐GFP vectors for subcellular analyses were constructed and injected into tobacco leaves using *Agrobacterium tumefaciens* strain GV3101‐mediated transformation. Additionally, 35S::TaPPR13‐nYFP, 35S::TaAOR1‐cYFP, and 35S::TaSIG5‐cYFP vectors were constructed for bimolecular fluorescence complementation (BiFC) assays, and the 35S::TaPPR13‐nLUC and 35S::TaAOR1‐cLUC vectors were constructed for luciferase complementation imaging (LCI) assays following previously described protocols.^[^ ^39^ ^]^ The co‐injection of constructed vectors (TaPPR13‐nYFP and TaAOR1‐cYFP, TaPPR13‐nYFP and TaSIG5‐cYFP, TaPPR13‐nLUC and TaAOR1‐cLUC) were performed in tobacco leaves as previously described.^[^ ^101^ ^]^ GFP and YFP fluorescence were visualized using a LSM900 confocal laser scanning microscope (Zeiss, Germany), and luciferase activity was detected using a Rocel In Vivo Plant Imaging system (BIOCOVER, Beijing). For LUC assays, the *TaPPR13* promoter was cloned into the pGreenII0800 vector to serve as the reporter and the 35S::TaBZR2‐GFP vector was used as an effector. LUC signals were detected using the Rocel In Vivo Plant Imaging system, and the LUC/REN ratio was quantified with using a Luciferase Assay Kit (Yeasen, Shanghai).

### Pull‐Down, EMSA and In Vitro Degradation Assays

Pull‐down assays were carried out to investigate interaction between TaPPR13 and TaAOR1. The recombinant fusion proteins TaPPR13‐His, TaAOR1‐GST, and TaSIG5‐GST were expressed in Transetta (DE3) cells (TransGen, Beijing) and purified using Ni‐NTA Resin (TransGen) and GST Resin (TransGen) beads, respectively. The pull‐down assay was then performed as previously described.^[^ ^102^ ^]^ TaBZR2‐GST recombinant fusion protein for EMSA assays was expressed and purified according to the manufacturer's instructions. DNA probes containing E‐box *cis*‐elements were incubated with the TaBZR2‐GST protein in EMSA buffer and EMSA was carried out using a LightShift EMSA kit (ThermoScientific, USA) following the manufacturer's protocol. Leaf‐proteins were extracted using plant total protein extract kit (BestBio, Shanghai) following the manufacturer's protocol, and in vitro degradation assays were carried out as previously described.^[^ ^6^ ^]^ For degradation assays of TaAOR1 and TaSIG5, equal amounts (c. 1 µg) of purified TaAOR1‐GST and TaSIG5‐GST were incubated in 20 µL total proteins (c. 350 µg) of WT, *TaPPR13*‐OE or *TaPPR13*‐KD plants. The mixtures were incubated at 25 °C for 30 min, and protein abundances of TaAOR1 and TaSIG5 were determined using immunoblot and anti‐GST antibody.

### Statistical Analysis

Statistical analyses were conducted using Microsoft Excel, where mean values and standard deviations (SD) were calculated. The significance of differences between two groups was determined using the Student's *t*‐test. Statistical comparisons among the mean values were performed using one‐way analysis of variance (ANOVA), followed by Tukey's multiple range test. *p*‐value of < 0.05 was considered statistically significant.

## Conflict of Interest

The authors declare no conflict of interest.

## Author Contributions

Z.‐H.H., W.‐J.Z., L.Z., and J.‐Y.W. contributed equally to this work. Z.‐S.X. conceived the study and designed the research. X.‐J. N. conducted the GWAS. Z.‐H. H., W.‐J. Z., L.Z., and J.‐Y.W. performed most of the experiments and analyzed the data. Z.‐H.H. wrote the article. S.‐X.Z., J.‐T.W., S.‐H.Y., Y.‐C.J., W.‐J.C., T.‐F.Y., X.‐F.M., J.‐N.R., Y.‐W.L., X.‐Y.C., J.C., Y.‐B.Z., M.C., and Y.‐Z.M. commented on the manuscript. L.‐H.L. provided the wheat seeds.

## Supporting information



Supporting Information

Supplemental Table 1

Supplemental Table 2

Supplemental Table 3

## Data Availability

The data that support the findings of this study are available from the corresponding author upon reasonable request.

## References

[1] J. Liu , Y. Yao , M. Xin , H. Peng , Z. Ni , Q. Sun , J. Integr. Plant Biol. 2022, 64, 536. 34962080 10.1111/jipb.13210

[2] S. Li , Y. Zhang , Y. Liu , P. Zhang , X. Wang , B. Chen , L. Ding , Y. Nie , F. Li , Z. Ma , Z. Kang , H. Mao , Plant Cell 2023, 36, 605. 10.1093/plcell/koad307 PMC10896296 38079275

[3] J. Ma , Y. Geng , H. Liu , M. Zhang , S. Liu , C. Hao , J. Hou , Y. Zhang , D. Zhang , W. Zhang , X. Zhang , T. Li , J. Integr. Plant Biol. 2023, 65, 2056. 37310066 10.1111/jipb.13542

[4] J. P. Li , X. B. Liu , S. M. Chang , W. Chu , J. C. Lin , H. Zhou , Z. R. Hu , M. C. Zhang , M. M. Xin , Y. Y. Yao , W. L. Guo , X. D. Xie , H. R. Peng , Z. F. Ni , Q. X. Sun , Y. Long , Z. R. Hu , Sci. Adv. 2024, 10, adk4027. 10.1126/sciadv.adk4027 PMC11014451 38608020

[5] Y. Song , L. Feng , M. A. M. Alyafei , A. Jaleel , M. Ren , Int. J. Mol. Sci. 2021, 22, 13464. 34948261 10.3390/ijms222413464 PMC8705820

[6] Y. Liu , T. F. Yu , Y. T. Li , L. Zheng , Z. W. Lu , Y. B. Zhou , J. Chen , M. Chen , J. P. Zhang , G. Z. Sun , X. Y. Cao , Y. W. Liu , Y. Z. Ma , Z. S. Xu , New Phytol. 2022, 236, 114. 35719110 10.1111/nph.18326 PMC9544932

[7] I. Serrano , C. Audran , S. Rivas , J. Exp. Bot. 2016, 67, 3845. 26994477 10.1093/jxb/erw088

[8] G. R. Littlejohn , S. Breen , N. Smirnoff , M. Grant , New Phytol. 2021, 229, 3088. 33206379 10.1111/nph.17076

[9] M. Li , C. Kim , Plant Commun. 2022, 3, 100264. 35059631 10.1016/j.xplc.2021.100264 PMC8760138

[10] L. Mignolet‐Spruyt , E. Xu , N. Idänheimo , F. A. Hoeberichts , P. Mühlenbock , M. Brosché , F. Van Breusegem , J. Kangasjärvi , J. Exp. Bot. 2016, 67, 3831. 26976816 10.1093/jxb/erw080

[11] S. Karpinski , C. Escobar , B. Karpinska , G. Creissen , P. M. Mullineaux , Plant Cell 1997, 9, 627. 9144965 10.1105/tpc.9.4.627 PMC156944

[12] L. Bai , P. Wang , C.‐P. Song , Reactive Oxygen Species (ROS) and ABA Signalling, Springer, Dordrecht 2014.

[13] C. H. Foyer , G. Hanke , Plant J. 2022, 111, 642. 35665548 10.1111/tpj.15856 PMC9545066

[14] D. Cho , D. Shin , B. W. Jeon , J. M. Kwak , J. Plant Biol. 2009, 52, 102.

[15] B. Castro , M. Citterico , S. Kimura , D. M. Stevens , M. Wrzaczek , G. Coaker , Nat. Plants 2021, 7, 403. 33846592 10.1038/s41477-021-00887-0 PMC8751180

[16] Y. Miao , D. Lv , P. Wang , X.‐C. Wang , J. Chen , C. Miao , C.‐P. Song , Plant Cell 2006, 18, 2749. 16998070 10.1105/tpc.106.044230 PMC1626619

[17] G. K. Rai , D. M. Khanday , S. M. Choudhary , P. Kumar , S. Kumari , C. Martínez‐Andújar , P. A. Martínez‐Melgarejo , P. K. Rai , F. Pérez‐Alfocea , Plant Stress 2024, 11, 100359.

[18] J.‐J. Zou , X.‐D. Li , D. Ratnasekera , C. Wang , W.‐X. Liu , L.‐F. Song , W.‐Z. Zhang , W.‐H. Wu , Plant Cell 2015, 27, 1445. 25966761 10.1105/tpc.15.00144 PMC4456645

[19] N. O'Toole , M. Hattori , C. Andres , K. Iida , C. Lurin , C. Schmitz‐Linneweber , M. Sugita , I. Small , Mol. Biol. Evol. 2008, 25, 1120. 18343892 10.1093/molbev/msn057

[20] L. Wu , J. Wu , Y. Liu , X. Gong , J. Xu , D. Lin , Y. Dong , Rice 2016, 9, 67. 27910002 10.1186/s12284-016-0134-1 PMC5133210

[21] H. Xiao , Z. Liu , X. Zou , Y. Xu , L. Peng , J. Hu , H. Lin , J. Plant Physiol. 2021, 258, 153361. 33429329 10.1016/j.jplph.2020.153361

[22] T. Qin , P. Zhao , J. Sun , Y. Zhao , Y. Zhang , Q. Yang , W. Wang , Z. Chen , T. Mai , Y. Zou , G. Liu , W. Hao , Front. Genet. 2021, 12, 765580. 34733319 10.3389/fgene.2021.765580 PMC8559896

[23] X. Wang , Y. An , Z. Qi , J. Xiao , Plant Sci. 2021, 308, 110908. 34034865 10.1016/j.plantsci.2021.110908

[24] Q. Zhang , C. Chen , Y. Wang , M. He , Z. Li , L. Shen , Q. Li , L. Zhu , D. Ren , J. Hu , Z. Gao , G. Zhang , Q. Qian , Plant Cell Rep. 2022, 42, 421. 36576552 10.1007/s00299-022-02968-6

[25] H. Higashi , Y. Kato , T. Fujita , S. Iwasaki , M. Nakamura , Y. Nishimura , M. Takenaka , T. Shikanai , Plant Cell Physiol. 2021, 62, 1146. 33439244 10.1093/pcp/pcaa180

[26] Y. Lv , Y. Wang , Q. Zhang , C. Chen , Q. Qian , L. Guo , Plant Sci. 2022, 323, 111382. 35850283 10.1016/j.plantsci.2022.111382

[27] H. Xiao , Q. Zhang , X. Qin , Y. Xu , C. Ni , J. Huang , L. Zhu , F. Zhong , W. Liu , G. Yao , Y. Zhu , J. Hu , New Phytol. 2018, 220, 878. 30019754 10.1111/nph.15347

[28] P. Zheng , Y. Liu , X. Liu , Y. Huang , F. Sun , W. Wang , H. Chen , M. Jan , C. Zhang , Y. Yuan , B.‐C. Tan , H. Du , J. Tu , Theor. Appl. Genet. 2021, 134, 923. 33386861 10.1007/s00122-020-03742-6 PMC7925476

[29] L. Tadini , C. Peracchio , A. Trotta , M. Colombo , I. Mancini , N. Jeran , A. Costa , F. Faoro , M. Marsoni , C. Vannini , E. M. Aro , P. Pesaresi , Plant J. 2019, 101, 1198. 31648387 10.1111/tpj.14585

[30] P. Pesaresi , C. Kim , Plant Cell Rep. 2019, 38, 819. 30671650 10.1007/s00299-019-02383-4

[31] K. Laluk , S. AbuQamar , T. Mengiste , Plant Physiol. 2011, 156, 2053. 21653783 10.1104/pp.111.177501 PMC3149943

[32] S.‐C. Jiang , C. Mei , S. Liang , Y.‐T. Yu , K. Lu , Z. Wu , X.‐F. Wang , D.‐P. Zhang , Plant Mol. Biol. 2015, 88, 369. 26093896 10.1007/s11103-015-0327-9 PMC4486114

[33] K. Lu , C. Li , J. Guan , W.‐H. Liang , T. Chen , Q.‐Y. Zhao , Z. Zhu , S. Yao , L. He , X.‐D. Wei , L. Zhao , L.‐H. Zhou , C.‐F. Zhao , C.‐L. Wang , Y.‐D. Zhang , Rice 2022, 15, 62. 36463341 10.1186/s12284-022-00608-x PMC9719575

[34] H.‐G. Su , B. Li , X.‐Y. Song , J. Ma , J. Chen , Y.‐B. Zhou , M. Chen , D.‐H. Min , Z.‐S. Xu , Y.‐Z. Ma , Int. J. Mol. Sci. 2019, 20, 5667.31726763

[35] X.‐Y. Cui , Y. Gao , J. Guo , T.‐F. Yu , W.‐J. Zheng , Y.‐W. Liu , J. Chen , Z.‐S. Xu , Y.‐Z. Ma , Plant Physiol. 2019, 180, 605. 30842265 10.1104/pp.19.00100 PMC6501075

[36] G.‐H. He , J.‐Y. Xu , Y.‐X. Wang , J.‐M. Liu , P.‐S. Li , M. Chen , Y.‐Z. Ma , Z.‐S. Xu , BMC Plant Biol. 2016, 16, 116. 27215938 10.1186/s12870-016-0806-4 PMC4877946

[37] X. Liu , J. Lan , Y. Huang , P. Cao , C. Zhou , Y. Ren , N. He , S. Liu , Y. Tian , T. Nguyen , L. Jiang , J. Wan , J. Exp. Bot. 2018, 69, 3949. 29893948 10.1093/jxb/ery214 PMC6054151

[38] K. Hwang , H. Susila , Z. Nasim , J.‐Y. Jung , J. H. Ahn , Mol. Plant 2019, 12, 489. 30639313 10.1016/j.molp.2019.01.002

[39] T. F. Yu , Y. Liu , J. D. Fu , J. Ma , Z. W. Fang , J. Chen , L. Zheng , Z. W. Lu , Y. B. Zhou , M. Chen , Z. S. Xu , Y. Z. Ma , Plant Biotechnol. J. 2021, 19, 2589. 34416065 10.1111/pbi.13684 PMC8633499

[40] Z. Hou , X. Zhang , Y. Tang , T. Yu , L. Zheng , J. Chen , Y. Zhou , Y. Liu , M. Chen , Z.‐S. Xu , Y. Ma , Crop J. 2022, 10, 1601.

[41] Y. Yamauchi , A. Hasegawa , M. Mizutani , Y. Sugimoto , FEBS Lett. 2012, 586, 1208. 22575657 10.1016/j.febslet.2012.03.013

[42] A. Nagashima , M. Hanaoka , T. Shikanai , M. Fujiwara , K. Kanamaru , H. Takahashi , K. Tanaka , Plant Cell Physiol. 2004, 45, 357. 15111710 10.1093/pcp/pch050

[43] D. L. Cano‐Ramirez , P. E. Panter , T. Takemura , T. S. de Fraine , L. L. de Barros Dantas , R. Dekeya , T. Barros‐Galvão , P. Paajanen , A. Bellandi , T. Batstone , B. F. Manley , K. Tanaka , S. Imamura , K. A. Franklin , H. Knight , A. N. Dodd , Nat. Plants 2023, 9, 661. 36997687 10.1038/s41477-023-01377-1 PMC10119024

[44] P. Albertos , G. Dündar , P. Schenk , S. Carrera , P. Cavelius , T. Sieberer , B. Poppenberger , EMBO J. 2022, 41, 108664. 10.15252/embj.2021108664 PMC8804921 34981847

[45] X. Y. Hao , T. F. Yu , C. J. Peng , Y. H. Fu , Y. H. Fang , Y. Li , Z. S. Xu , J. Chen , H. B. Dong , Y. Z. Ma , W. G. Xu , Plant Biotechnol. J. 2025, doi:10.1111/pbi.70045. 10.1111/pbi.70045 PMC12205877 40183279

[46] W. Chu , S. Chang , J. Lin , C. Zhang , J. Li , X. Liu , Z. Liu , D. Liu , Q. Yang , D. Zhao , X. Liu , W. Guo , M. Xin , Y. Yao , H. Peng , C. Xie , Z. Ni , Q. Sun , Z. Hu , Plant Cell 2024, 36, 2607. 38537937 10.1093/plcell/koae100 PMC11218785

[47] X. Wang , X. Chen , Q. Wang , M. Chen , X. Liu , D. Gao , D. Li , L. Li , Front. Plant Sci. 2019, 10, 1473. 31827478 10.3389/fpls.2019.01473 PMC6892407

[48] C. Jia , S. Zhao , T. Bao , P. Zhao , K. Peng , Q. Guo , X. Gao , J. Qin , Plant Sci. 2021, 302, 110719. 33288025 10.1016/j.plantsci.2020.110719

[49] R. Yang , Z. Yang , M. Xing , Y. Jing , Y. Zhang , K. Zhang , Y. Zhou , H. Zhao , W. Qiao , J. Sun , J. Genet. Genom. 2023, 50, 861. 10.1016/j.jgg.2023.09.006 37734712

[50] Q. Li , F. Xu , Z. Chen , Z. Teng , K. Sun , X. Li , J. Yu , G. Zhang , Y. Liang , X. Huang , L. Du , Y. Qian , Y. Wang , C. Chu , J. Tang , Nat. Plants 2021, 7, 1108. 34226689 10.1038/s41477-021-00959-1

[51] J. Zhou , J. Wang , X. Li , X.‐J. Xia , Y.‐H. Zhou , K. Shi , Z. Chen , J.‐Q. Yu , J. Exp. Bot. 2014, 65, 4371. 24899077 10.1093/jxb/eru217 PMC4112640

[52] A. Zhang , J. Zhang , J. Zhang , N. Ye , H. Zhang , M. Tan , M. Jiang , Plant Cell Physiol. 2010, 52, 181. 21134899 10.1093/pcp/pcq187

[53] S. An , Y. Liu , K. Sang , T. Wang , J. Yu , Y. Zhou , X. Xia , J. Integr. Plant Biol. 2023, 65, 10. 36053143 10.1111/jipb.13356

[54] A. Barkan , I. Small , Annu. Rev. Plant Biol. 2014, 65, 415. 24471833 10.1146/annurev-arplant-050213-040159

[55] H. Xing , X. Fu , C. Yang , X. Tang , L. Guo , C. Li , C. Xu , K. Luo , Sci. Rep. 2018, 8, 2817. 29434322 10.1038/s41598-018-21269-1 PMC5809412

[56] X. Li , M. Sun , S. Liu , Q. Teng , S. Li , Y. Jiang , Int. J. Mol. Sci. 2021, 22, 11274. 34681932 10.3390/ijms222011274 PMC8537650

[57] L. Meng , M. Du , T. Zhu , G. Li , Y. Ding , Q. Zhang , Front. Plant Sci. 2024, 15, 1416742. 38993942 10.3389/fpls.2024.1416742 PMC11236678

[58] X. Zu , L. Luo , Z. Wang , J. Gong , C. Yang , Y. Wang , C. Xu , X. Qiao , X. Deng , X. Song , C. Chen , B.‐C. Tan , X. Cao , Nat. Commun. 2023, 14, 6789. 37880207 10.1038/s41467-023-42269-4 PMC10600133

[59] H. Emami , F. Kempken , Plant J. 2019, 100, 265. 31219634 10.1111/tpj.14441

[60] K. J. Dietz , I. Turkan , A. Krieger‐Liszkay , Plant Physiol. 2016, 171, 1541. 27255485 10.1104/pp.16.00375 PMC4936569

[61] J. i. Mano , M. S. Biswas , K. Sugimoto , Plants 2019, 8, 391. 31575078 10.3390/plants8100391 PMC6843276

[62] T. Yalcinkaya , B. Uzilday , R. Ozgur , I. Turkan , J. i. Mano , Environ. Exp. Bot. 2019, 165, 139.

[63] P. Wang , W. C. Liu , C. Han , S. Wang , M. Y. Bai , C. P. Song , J. Integr. Plant Biol. 2024, 66, 330. 38116735 10.1111/jipb.13601

[64] K. Shah , S. Nahakpam , Plant Physiol. Biochem. 2012, 57, 106. 22698753 10.1016/j.plaphy.2012.05.007

[65] H. Yan , Q. Li , S.‐C. Park , X. Wang , Y.‐j. Liu , Y.‐g. Zhang , W. Tang , M. Kou , D.‐f. Ma , Plant Physiol. Biochem. 2016, 109, 20. 27620271 10.1016/j.plaphy.2016.09.003

[66] H. Yang , D. Zhang , H. Li , L. Dong , H. Lan , Plant Physiol. Biochem. 2015, 95, 83. 26202169 10.1016/j.plaphy.2015.07.001

[67] H. Yang , D. Zhang , X. Li , H. Li , D. Zhang , H. Lan , A. J. Wood , J. Wang , Mol. Breed. 2016, 36, 34.

[68] G. Tian , S. Wang , J. Wu , Y. Wang , X. Wang , S. Liu , D. Han , G. Xia , M. Wang , Nat. Commun. 2023, 14, 1200. 36864053 10.1038/s41467-023-36901-6 PMC9981739

[69] Y. Li , S. Han , X. Sun , N. U. Khan , Q. Zhong , Z. Zhang , H. Zhang , F. Ming , Z. Li , J. Li , J. Integr. Plant Biol. 2023, 65, 918. 36401566 10.1111/jipb.13414

[70] H. Gao , J. Cui , S. Liu , S. Wang , Y. Lian , Y. Bai , T. Zhu , H. Wu , Y. Wang , S. Yang , X. Li , J. Zhuang , L. Chen , Z. Gong , F. Qin , Mol. Plant 2022, 15, 1558. 36045577 10.1016/j.molp.2022.08.009

[71] T. F. Yu , Z. H. Hou , H. L. Wang , S. Y. Chang , X. Y. Song , W. J. Zheng , L. Zheng , J. T. Wei , Z. W. Lu , J. Chen , Y. B. Zhou , M. Chen , S. L. Sun , Q. Y. Jiang , L. G. Jin , Y. Z. Ma , Z. S. Xu , Plant Biotechnol. J. 2024, 22, 2333. 38600703 10.1111/pbi.14349 PMC11258977

[72] A.‐Z. Sun , F.‐Q. Guo , Front. Plant Sci. 2016, 7, 398. 27066042 10.3389/fpls.2016.00398 PMC4814484

[73] T. Crawford , N. Lehotai , Å. Strand , J. Exp. Bot. 2018, 69, 2783. 29281071 10.1093/jxb/erx481

[74] A. S. Richter , T. Nägele , B. Grimm , K. Kaufmann , M. Schroda , D. Leister , T. Kleine , Plant Commun. 2023, 4, 100511. 36575799 10.1016/j.xplc.2022.100511 PMC9860301

[75] F. Gao , J. Guo , Y. Shen , Plant Stress 2024, 12, 100470.

[76] C. Zhao , A. Haigh , P. Holford , Z.‐H. Chen , Int. J. Mol. Sci. 2018, 19, 963. 29570668 10.3390/ijms19040963 PMC5979362

[77] Y. Zhuang , M. Wei , C. Ling , Y. Liu , A. K. Amin , P. Li , P. Li , X. Hu , H. Bao , H. Huo , J. Smalle , S. Wang , Cell Rep. 2021, 36, 109384. 34260941 10.1016/j.celrep.2021.109384

[78] L. Zsigmond , G. b. Rigó , A. s. Szarka , G. n. Székely , K. Ötvös , Z. Darula , K. F. Medzihradszky , C. Koncz , Z. Koncz , L. s. Szabados , Plant Physiol. 2008, 146, 1721. 18305213 10.1104/pp.107.111260 PMC2287346

[79] Y. Liu , J. He , Z. Chen , X. Ren , X. Hong , Z. Gong , Plant J. 2010, 63, 749. 20561255 10.1111/j.1365-313X.2010.04280.x

[80] J.‐M. Liu , J.‐Y. Zhao , P.‐P. Lu , M. Chen , C.‐H. Guo , Z.‐S. Xu , Y.‐Z. Ma , Front. Plant Sci. 2016, 7, 1825. 27994613 10.3389/fpls.2016.01825 PMC5136568

[81] W. Li , M. Deng , S. Wang , C. Wang , M. Guo , Y. Song , J. Guo , J. Yan , F. Ma , Q. Guan , J. Xu , Plant Physiol. 2023, 193, 2711. 37607253 10.1093/plphys/kiad468 PMC10663142

[82] M. Sierla , C. Waszczak , T. Vahisalu , J. Kangasjärvi , Plant Physiol. 2016, 171, 1569. 27208297 10.1104/pp.16.00328 PMC4936562

[83] C. Waszczak , M. Carmody , J. Kangasjärvi , Annu. Rev. Plant Biol. 2018, 69, 209. 29489394 10.1146/annurev-arplant-042817-040322

[84] Y. J. Yang , M. H. Bi , Z. F. Nie , H. Jiang , X. D. Liu , X. W. Fang , T. J. Brodribb , New Phytol. 2021, 230, 2001. 33586157 10.1111/nph.17278

[85] D. Luo , J. Liu , Y. Wu , X. Zhang , Q. Zhou , L. Fang , Z. Liu , Plant J. 2022, 112, 429. 36006043 10.1111/tpj.15955

[86] B. Ma , J. Zhang , S. Guo , X. Xie , L. Yan , H. Chen , H. Zhang , X. Bu , L. Zheng , Y. Wang , Hortic. Res. 2024, 11, uhae001. 38419969 10.1093/hr/uhae001 PMC10901477

[87] R. Hedrich , S. Shabala , Curr. Opin. Plant Biol. 2018, 46, 87. 30138845 10.1016/j.pbi.2018.07.015

[88] C. Shen , Y. Zhang , Q. Li , S. Liu , F. He , Y. An , Y. Zhou , C. Liu , W. Yin , X. Xia , New Phytol. 2021, 230, 1868. 33629353 10.1111/nph.17301

[89] S. Zhao , H. Gao , X. Jia , K. Zhou , H. Wang , K. Mao , F. Ma , Sci. Hortic. 2022, 294, 110758.

[90] J. Li , Y. Li , Z. Yin , J. Jiang , M. Zhang , X. Guo , Z. Ye , Y. Zhao , H. Xiong , Z. Zhang , Y. Shao , C. Jiang , H. Zhang , G. An , N. C. Paek , J. Ali , Z. Li , Plant Biotechnol. J. 2016, 15, 183. 27420922 10.1111/pbi.12601 PMC5258865

[91] G. B. Bhaskara , J. R. Lasky , S. Razzaque , L. Zhang , T. Haque , J. E. Bonnette , G. Z. Civelek , P. E. Verslues , T. E. Juenger , Proc. Natl. Acad. Sci. 2022, 119, 2205305119. 10.1073/pnas.2205305119 PMC9388090 35947617

[92] X. D. Li , Y. Q. Gao , W. H. Wu , L. M. Chen , Y. Wang , Plant Biotechnol. J. 2021, 20, 143. 34498364 10.1111/pbi.13701 PMC8710898

[93] J. Chen , Y. Gong , Y. Gao , Y. Zhou , M. Chen , Z. Xu , C. Guo , Y. Ma , Crop J. 2021, 9, 785.

[94] S. Chang , Q. Yang , W. Chu , X. Liu , J. Li , Z. Liu , J. Lin , D. Liu , D. Zhao , X. Peng , M. Xin , Y. Yao , X. Xie , H. Peng , Z. Ni , Q. Sun , Z. Hu , Plant Biotechnol. J. 2025, 23, 1650. 39977256 10.1111/pbi.14613 PMC12018820

[95] J. D. Woodson , J. M. Perez‐Ruiz , R. J. Schmitz , J. R. Ecker , J. Chory , Plant J. 2012, 73, 1. 22950756 10.1111/tpj.12011 PMC3605210

[96] P. Zhao , R. Cui , P. Xu , J. Wu , J.‐L. Mao , Y. Chen , C.‐Z. Zhou , L.‐H. Yu , C.‐B. Xiang , Sci. Rep. 2017, 7, 45492. 28358040 10.1038/srep45492 PMC5371990

[97] X. Wang , H. Wang , S. Liu , A. Ferjani , J. Li , J. Yan , X. Yang , F. Qin , Nat. Genet. 2016, 48, 1233. 27526320 10.1038/ng.3636

[98] S. Brunner , S. Hurni , G. Herren , O. Kalinina , S. von Burg , S. L. Zeller , B. Schmid , M. Winzeler , B. Keller , Plant Biotechnol. J. 2011, 9, 897. 21438988 10.1111/j.1467-7652.2011.00603.x

[99] R. Mega , F. Abe , J.‐S. Kim , Y. Tsuboi , K. Tanaka , H. Kobayashi , Y. Sakata , K. Hanada , H. Tsujimoto , J. Kikuchi , S. R. Cutler , M. Okamoto , Nat. Plants 2019, 5, 153. 30737511 10.1038/s41477-019-0361-8

[100] J. C. Isner , A. Begum , T. Nuehse , A. M. Hetherington , F. J. M. Maathuis , Curr. Biol. 2018, 28, 466. 29395926 10.1016/j.cub.2017.12.046

[101] Z.‐H. Hou , Y. Gao , J.‐C. Zheng , M.‐J. Zhao , Y. Liu , X.‐Y. Cui , Z.‐Y. Li , J.‐T. Wei , T.‐F. Yu , L. Zheng , Y.‐C. Jiao , S.‐H. Yang , J.‐M. Hao , J. Chen , Y.‐B. Zhou , M. Chen , L. Qiu , Y.‐Z. Ma , Z.‐S. Xu , J. Adv. Res. 2024, 3, S2090123224003874.

[102] D. Hu , R. Cui , K. Wang , Y. Yang , R. Wang , H. Zhu , M. He , Y. Fan , L. Wang , L. Wang , S. Chu , J. Zhang , S. Zhang , Y. Yang , X. Zhai , H. Lü , D. Zhang , J. Wang , F. Kong , D. Yu , H. Zhang , D. Zhang , Plant Cell 2024, 36, 2176. 38345432 10.1093/plcell/koae041 PMC11132883

