# Supplementary material for: TaPPR13, a Pentatricopeptide Repeat Protein Gene Activated by TaBZR2, Confers Drought Stress Tolerance by Enhancing the Antioxidant Defense System and Promoting Retrograde Signaling in Wheat (Triticum aestivum)
Source: Adv Sci (Weinh). 2025 Jun 29;12(36):e02984. doi: 10.1002/advs.202502984 (PMC12462999; doi:10.1002/advs.202502984)
Supplement: Supplementary file 1 — Supporting Information [file ADVS-12-e02984-s003.docx]

**Figure legends**

**
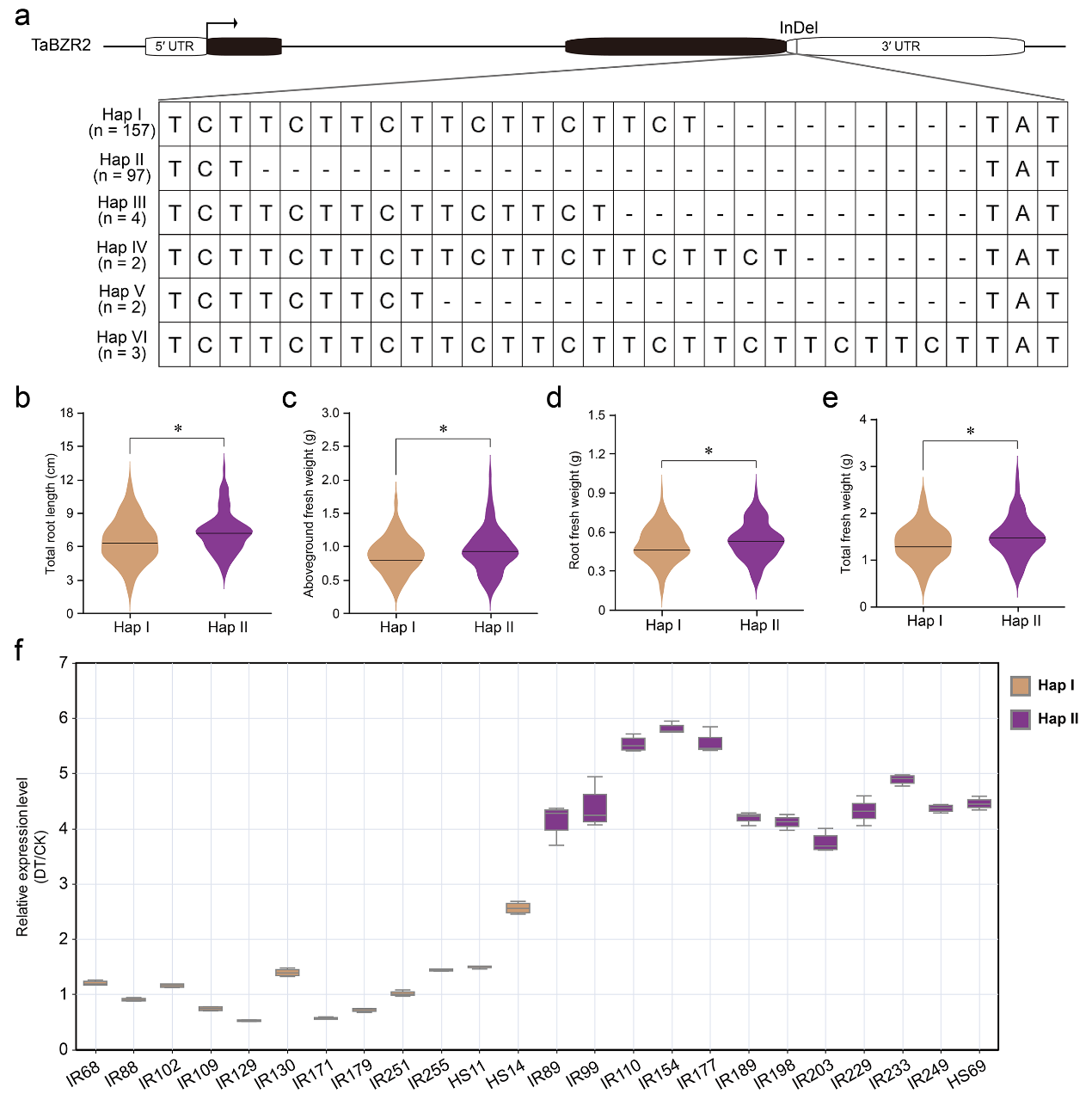
**

**Figure S1. Haplotype analysis of *TaBZR2*. (a) Gene structure and haplotypes of *TaBZR2*. (b-e) Total root length (b), aboveground fresh weight (c), root fresh weight (d), and total fresh weight (e) of accessions carrying *TaBZR2*-Hap I or *TaBZR2*-Hap II haplotypes. Values are means ± SD (*p* < 0.05, n = 157 for *TaBZR2*-Hap I haplotypes, n = 97 for *TaBZR2*-Hap II haplotypes, Student’s t-test). (f) The expression levels of *TaBZR2* carrying *TaBZR2*-Hap I or *TaBZR2*-Hap II haplotypes. Values are means ± SD (n = 4).**

**
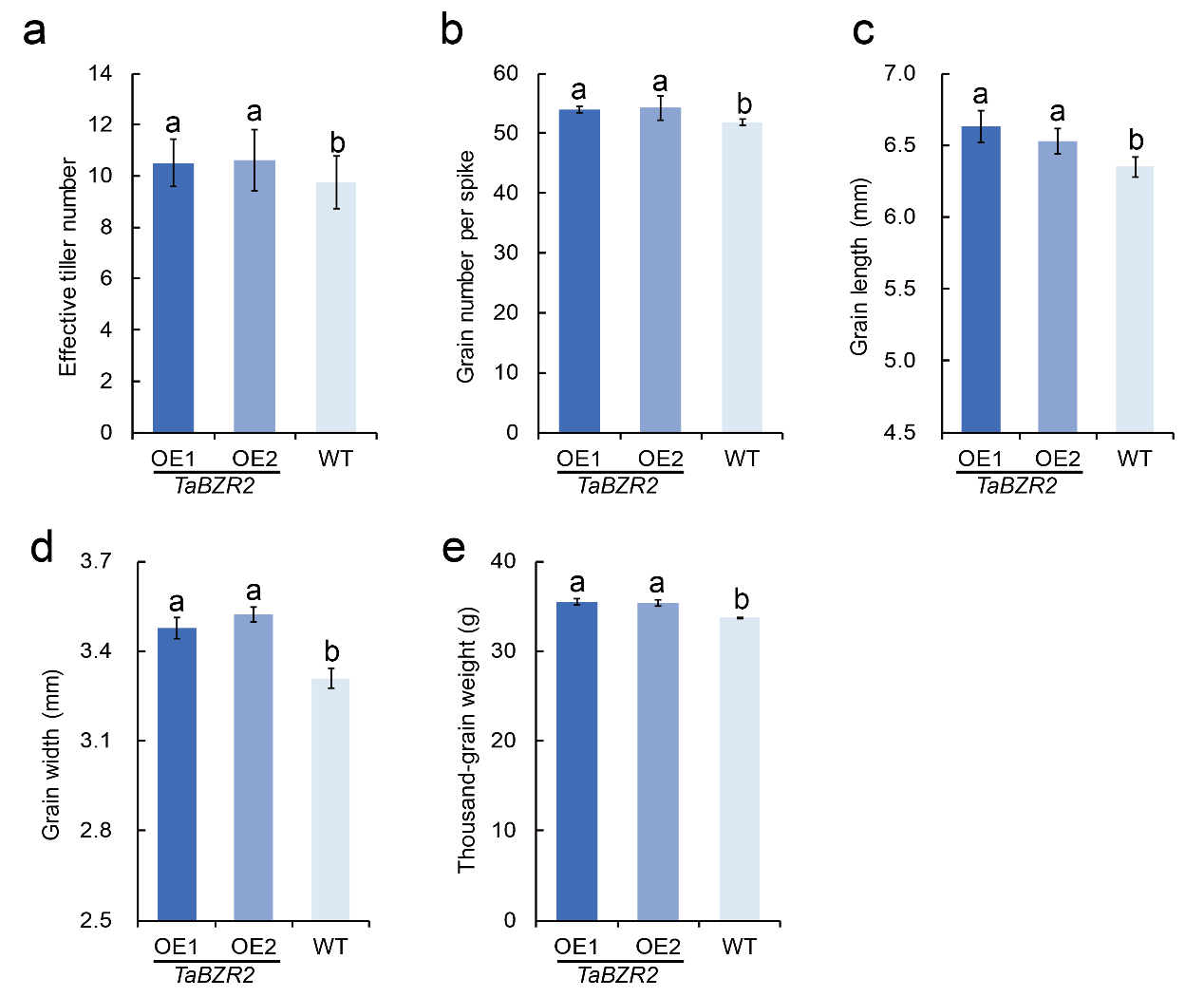
**

**Figure S2. Statistical data for effective tiller number (a), grain number per spike (b), grain length (c), grain width (d), and thousand kernel weight (e) of WT and *TaBZR2*-OE wheat plants grown under drought stress conditions. Values are means ± SD from three independent experiments (n > 30, *p* < 0.05, one way ANOVA, Tukey’s HSD test).**

**
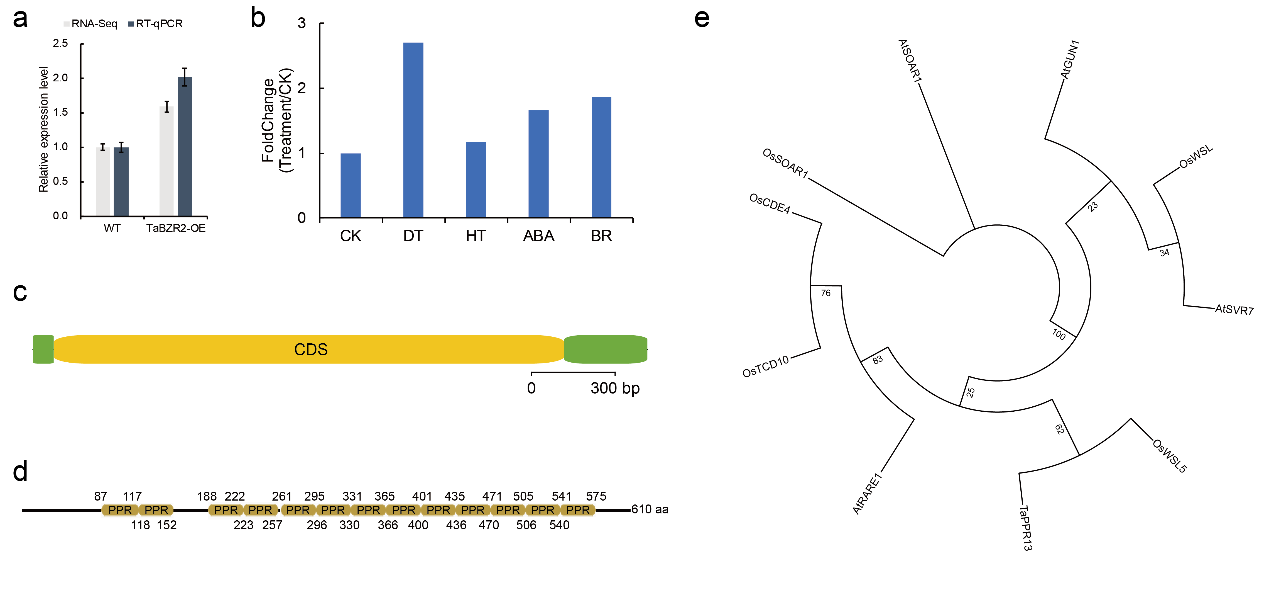
**

**Figure S3. Sequence and expression analysis of TaPPR13. (a) Comparative RNA-seq analysis showed high transcription levels of TaPPR13 in *TaBZR2*-OE plants under drought stress. Values are means ± SD (n = 3). (b) Comparative RNA-seq analysis showed high transcription levels of *TaPPR13* under drought stress, as well as during ABA and BR treatments. (c-d) Genomic structure (c), protein sequence (d) and phylogenetic analysis (e) of TaPPR13.**


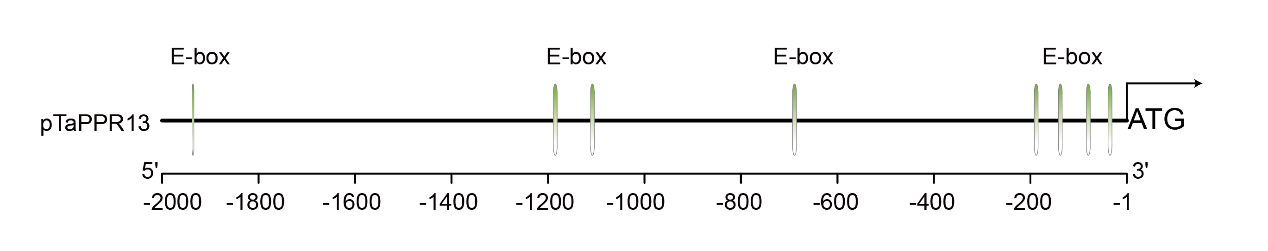


**Figure S4. Schematic of E-box *cis*-elements in the *TaPPR13* promoter.**

**
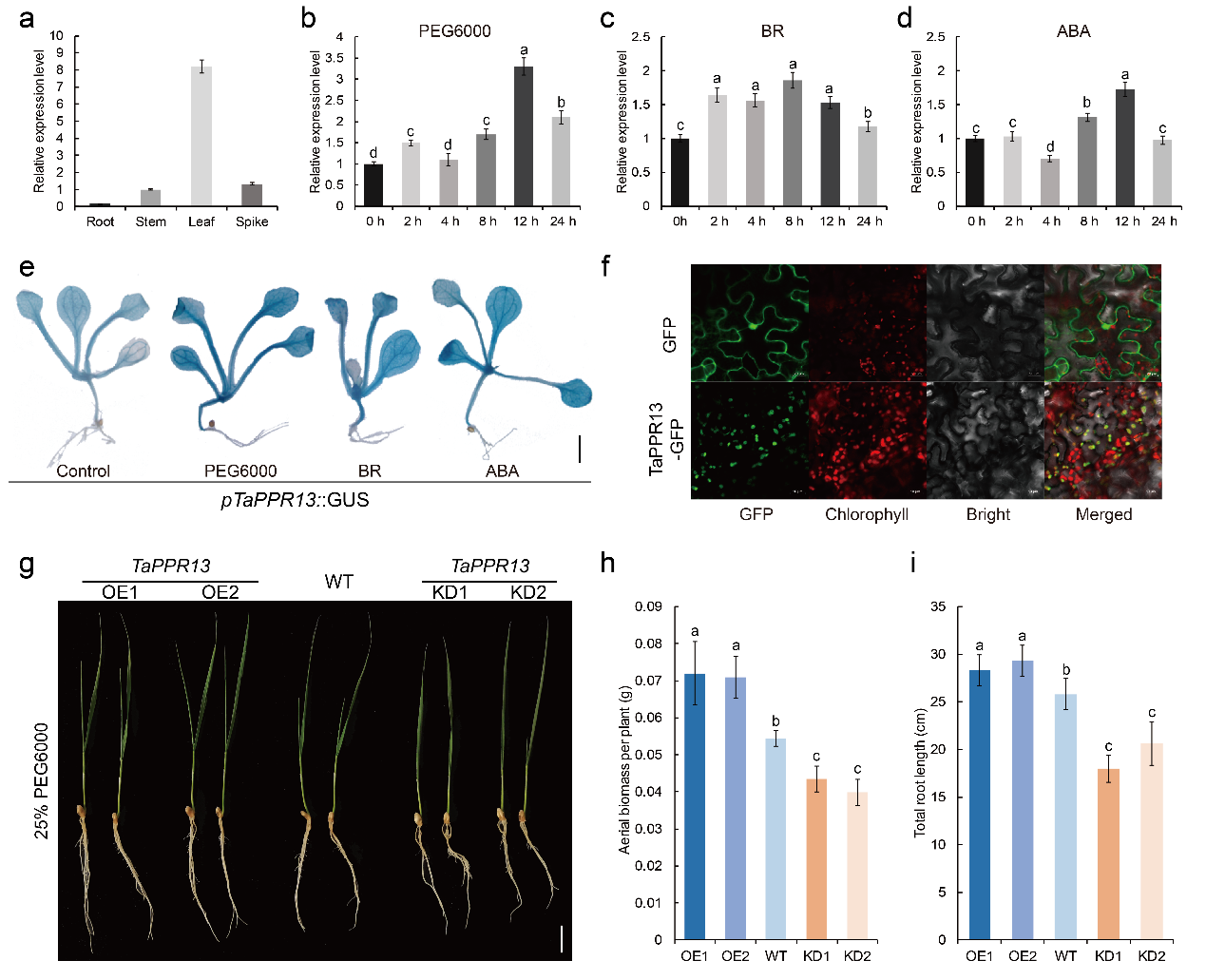
**

**Figure S5. TaPPR13 is a positive regulator of drought stress tolerance. (a) Tissue-specific expression patterns of the *TaPPR13* gene. Values are means ± SD (n = 4). (b-d) Expression patterns of the *TaPPR13* gene following drought stress (PEG6000), BR, and ABA treatments. Values are means ± SD (n = 4, *p* < 0.05, one way ANOVA, Tukey’s HSD test). (e) GUS assay of *proTaPPR13*::GUS transgenic plants following PEG6000, BR, and ABA treatment; Bar = 1 cm. (f) Subcellular localization of the TaPPR13-GFP protein; (g)** **Phenotypes of *TaPPR13*-OE, wild-type (WT), and *TaPPR13*-KD plants under simulated drought stress using PEG6000; Bar = 2 cm. (h, i) Mean aerial biomass (h) and total root length (i) of *TaPPR13*-OE, wild-type (WT), and *TaPPR13*-KD plants. Values are means ± SD (*p* < 0.05, n = 8, one way ANOVA, Tukey’s HSD test).**

**
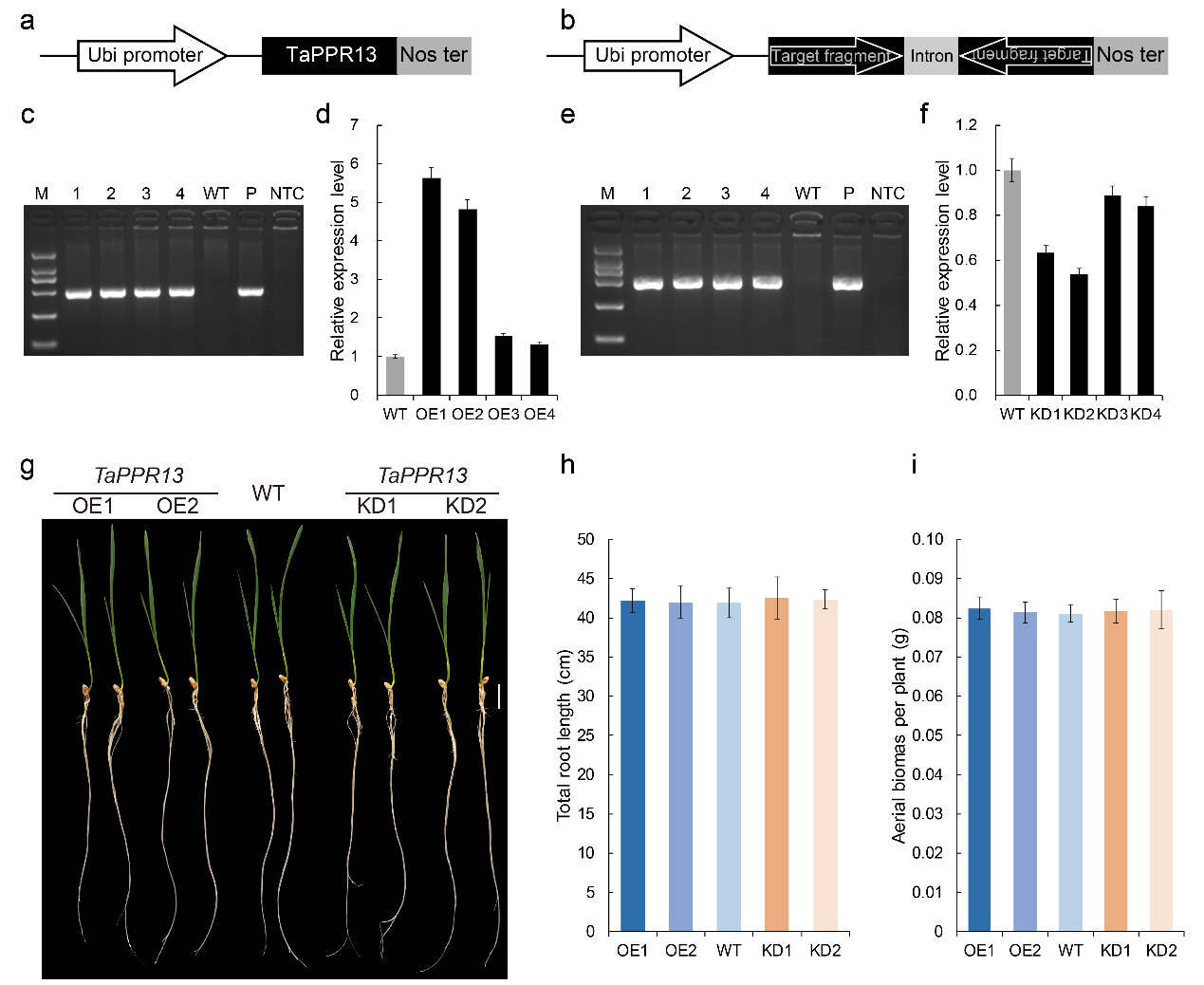
**

**Figure S6. Generation of *TaPPR13*-OE and *TaPPR13*-KD wheat plants. (a, b) Schematics of the structures of the overexpression (a) and knockdown (b) vectors. (c-f) Identification of *TaPPR13*-OE and *TaPPR13*-KD plants using PCR and qPCR. Values are means ± SD (n = 4). (g) Phenotypic analysis of *TaPPR13*-OE, WT, and *TaPPR13*-KD plants under controlled conditions; Bar = 2 cm. (h, i) Total root length (h) and aerial biomass (i) of *TaPPR13*-OE, WT, and *TaPPR13*-KD plants. Values are means ± SD (n = 8).**

**
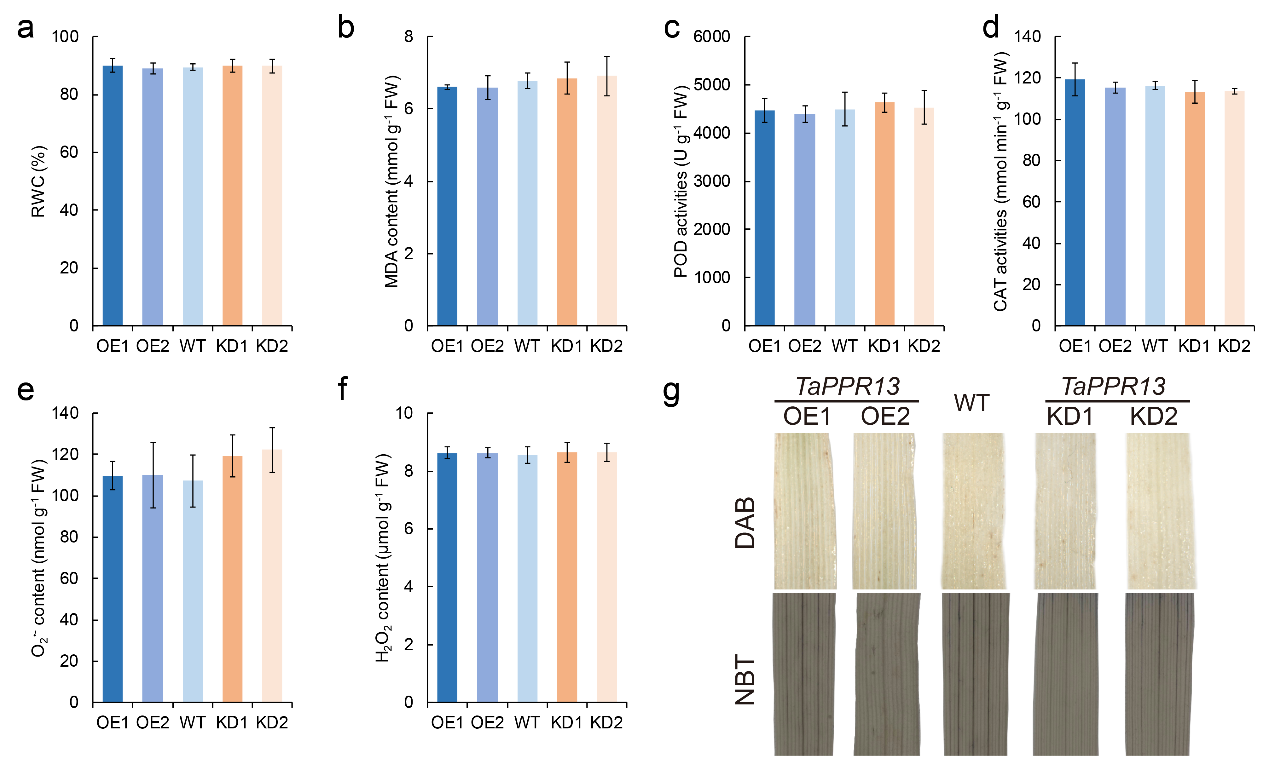
**

**Figure S7. Analysis of physiological indices in *TaPPR13*-OE, WT and *TaPPR13*-KD plants under normal conditions. (a) Relative water content (RWC) of *TaPPR13*-OE, WT, and *TaPPR13*-KD plants under normal conditions. Values are means ± SD (n = 3). (b) MDA content of *TaPPR13*-OE, WT, and *TaPPR13*-KD plants. Values are means ± SD (n = 3). (c, d) The POD and CAT activities of *TaPPR13*-OE, WT, and *TaPPR13*-KD plants. Values are means ± SD (n = 3). (e, f) O_2_^🞄-^ and H_2_O_2_ content of *TaPPR13*-OE, WT, and *TaPPR13*-KD plants. Values are means ± SD (n = 3). (g) DAB and NBT staining of *TaPPR13*-OE, WT, and *TaPPR13*-KD plants under normal conditions.**

**
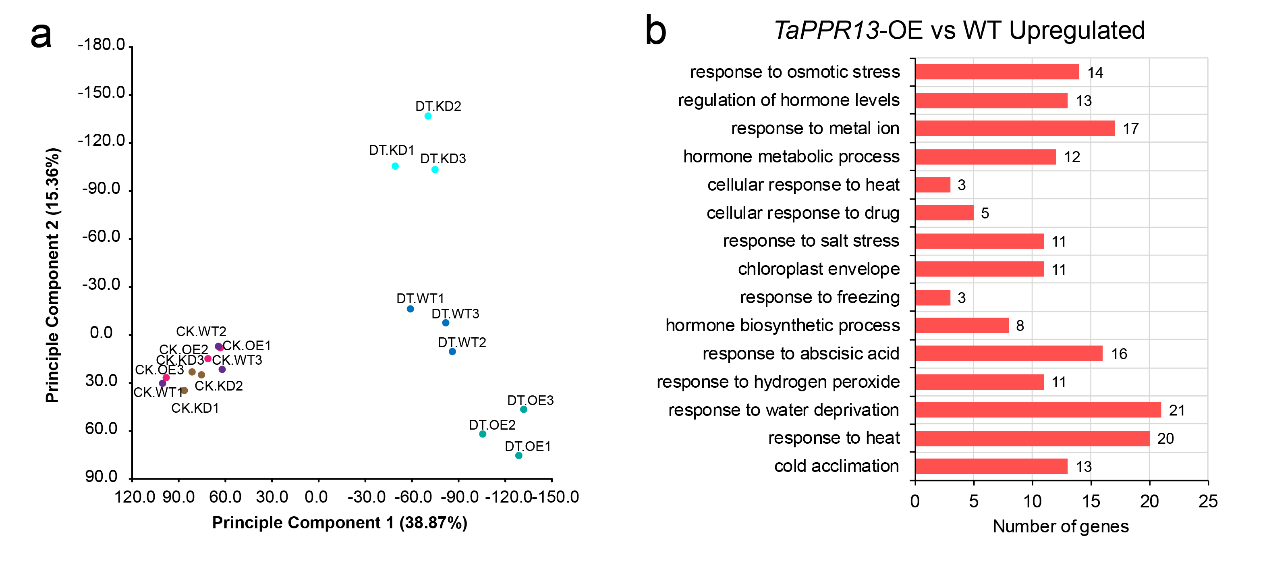
**

**Figure S8. Comparative RNA-seq analysis of *TaPPR13*-OE, WT, and *TaPPR13*-KD plants. (a) Principal component analysis (PCA) of the samples from *TaPPR13*-OE, WT, and *TaPPR13*-KD plants. (b) GO enrichment analysis of upregulated DEGs in *TaPPR13*-OE plants.**

**
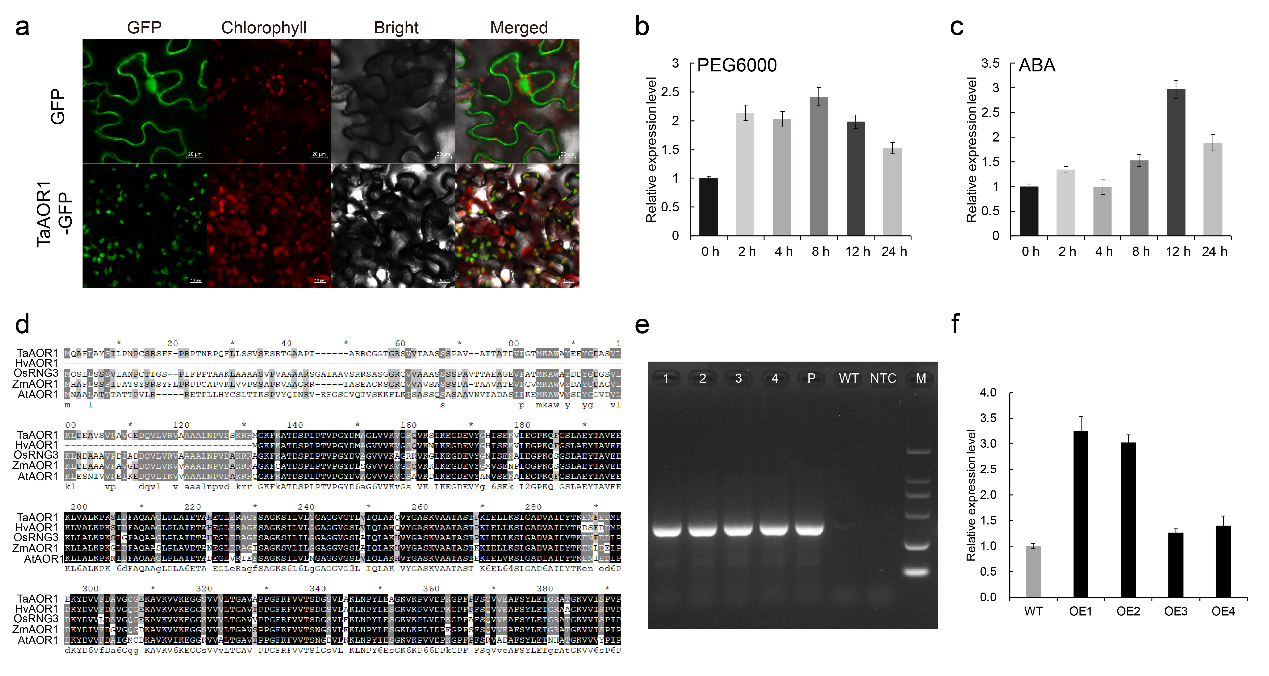
**

**Figure S9. Bioinformatics and expression pattern analysis of TaAOR1 (TraesCS6A02G133100). (a) Subcellular localization of the TaAOR1-GFP protein. (b, c) Expression patterns of the *TaAOR1* gene under drought stress (b) and ABA (c) treatment. Values are means ± SD (n = 4). (d) Amino acid sequence alignment of AOR1 proteins. (e, f) Identification of *TaAOR1*-OE wheat plants by PCR and qPCR. Values are means ± SD (n = 4).**

**
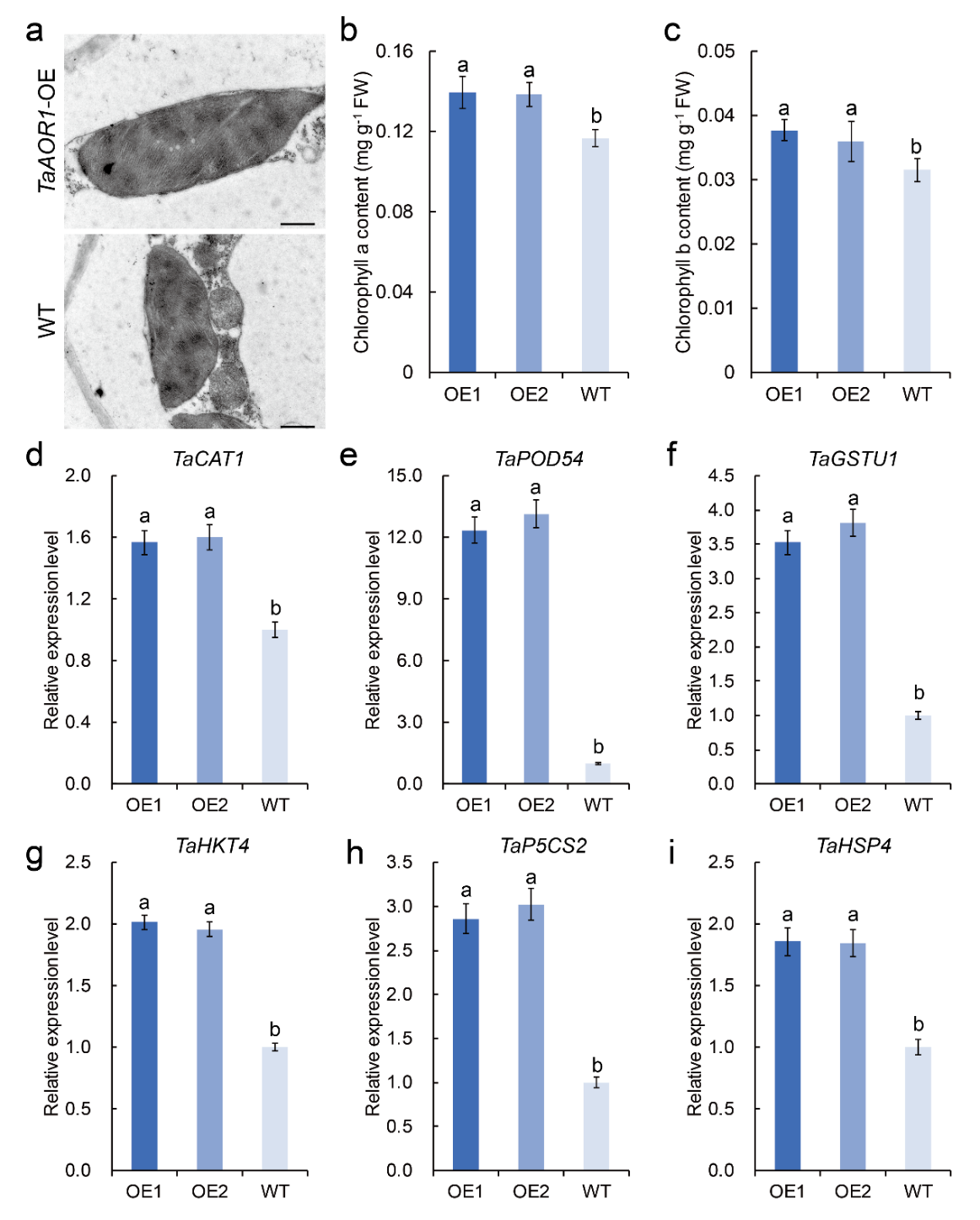
**

**Figure S10. TaAOR1 protects the chloroplasts in wheat. (a) TEM images of chloroplast ultrastructure in WT and *TaAOR1*-OE plants. (b, c) Chlorophyll a (b) and b (c) contents of *TaAOR1*-OE and WT plants under drought stress.** **Values are means ± SD (*p* < 0.05, n = 3, one way ANOVA, Tukey’s HSD test). (d-i) RT-qPCR analysis of the expression patterns of abiotic-stress response genes between WT and *TaAOR1*-OE plants under water deficit conditions.** **Values are means ± SD (*p* < 0.05, n = 4, one way ANOVA, Tukey’s HSD test).**


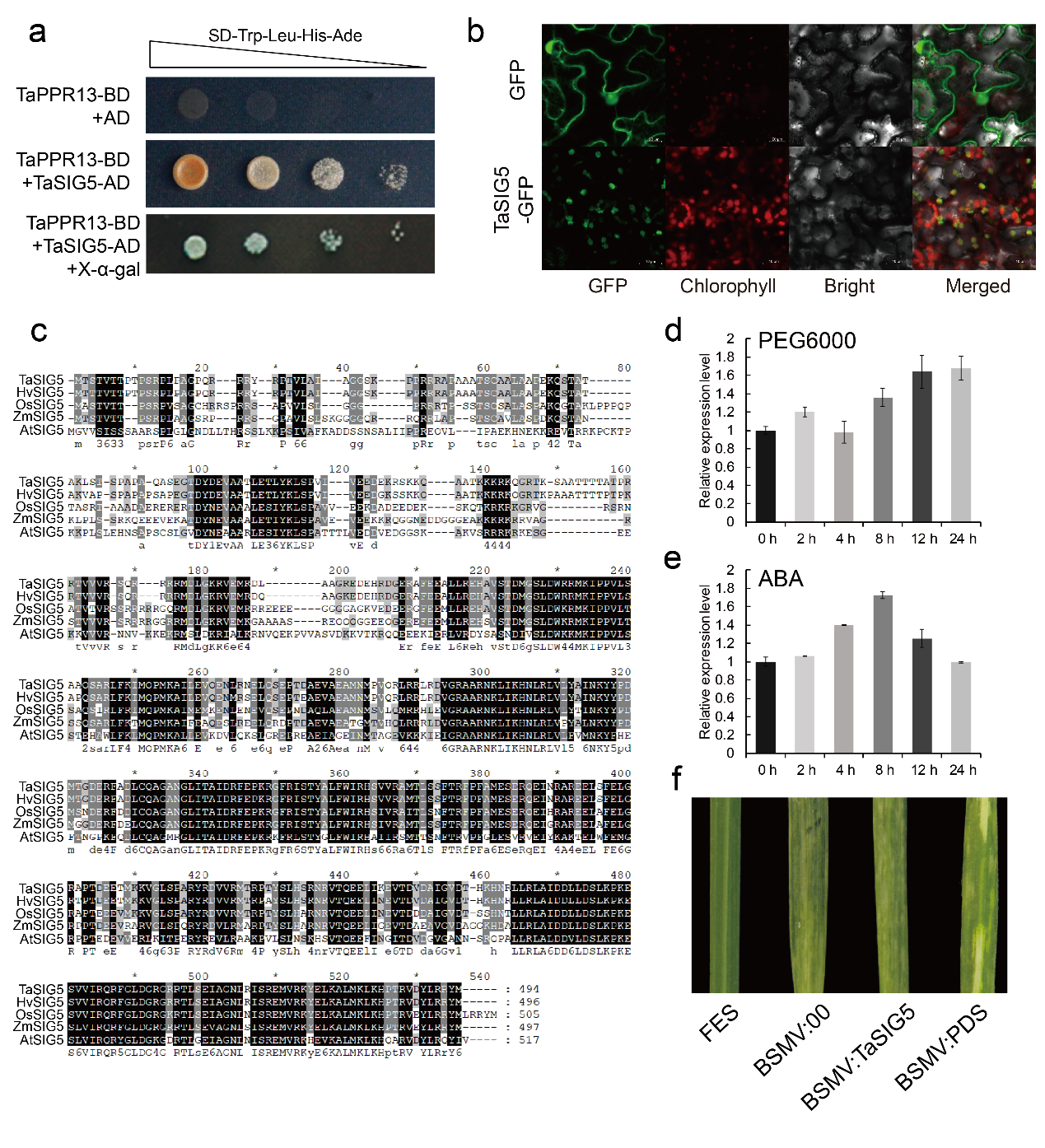


**Figure S11. Bioinformatics and expression pattern analysis of TaSIG5 (TraesCS1D02G436500). (a) Interaction between TaPPR13 and TaSIG5 proteins verified by yeast two-hybrid assay. (b) Subcellular localization of the TaSIG5-GFP protein. (c) Amino acid sequence alignment of SIG5 proteins. (d, e) Expression patterns of the *TaSIG5* gene under drought stress (d) and ABA (e) treatments. Values are means ± SD (n = 4). (f) Wheat leaves (cv. Pubingzi300) treated with FES buffer, BSMV:00 (γ empty vector), BSMV:TaSIG5, or BSMV:PDS (phytoene desaturase) for BSMV knockdown.**


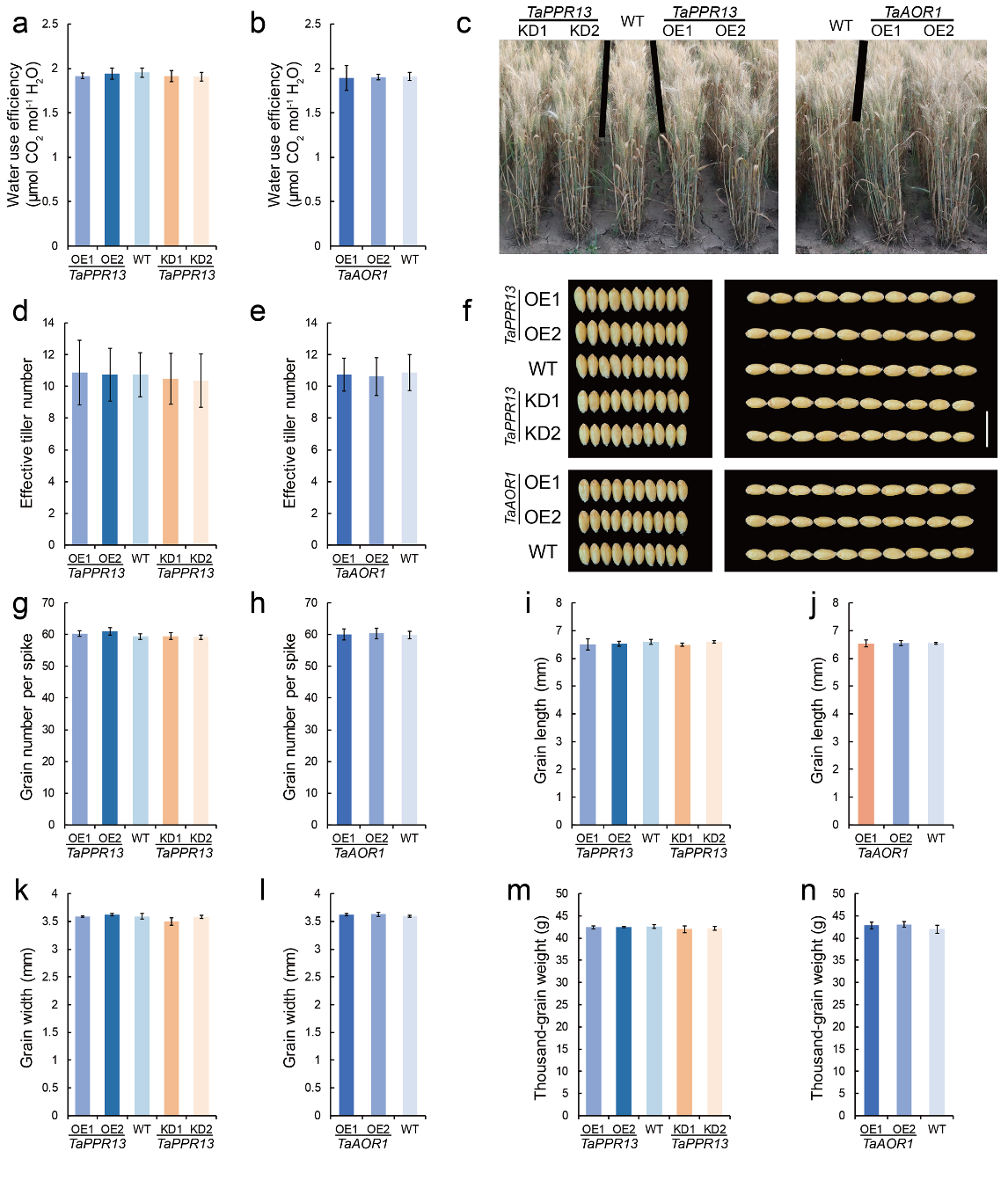


**Figure S12. Water use efficiency and yield per plant of transgenic wheat under normal field conditions. (a, b) Water use efficiency of WT and transgenic wheat under normal conditions. (c) Phenotypes of WT and transgenic wheat grown under normal field conditions. (d, e) Statistical data for effective tiller number of WT and transgenic wheat plants grown under normal field condition. Values are means ± SD (n = 8). (f) Grain width and length phenotypes of transgenic wheat and WT plants; Bar = 1 cm. (g-n) Statistical data for grain number per spike (g, h), grain length (i, j), grain width (k, l), and thousand kernel weight (m, n) of WT and transgenic wheat plants grown under normal field conditions.** **Values are means ± SD from three independent experiments (n > 30).**
